# The *Vibrio cholerae var* regulon encodes a metallo-β-lactamase and an antibiotic efflux pump, which are regulated by VarR, a LysR-type transcription factor

**DOI:** 10.1371/journal.pone.0184255

**Published:** 2017-09-12

**Authors:** Hong-Ting Victor Lin, Teresa Massam-Wu, Chen-Ping Lin, Yen-Jen Anna Wang, Yu-Chi Shen, Wen-Jung Lu, Pang-Hung Hsu, Yu-Hou Chen, Maria Ines Borges-Walmsley, Adrian Robert Walmsley

**Affiliations:** 1 Department of Food Science, National Taiwan Ocean University, Keelung, Taiwan; 2 Center of Excellence for the Oceans, National Taiwan Ocean University, Keelung, Taiwan; 3 Department of Biosciences, Durham University, Durham, United Kingdom; 4 Department of Bioscience and Biotechnology, National Taiwan Ocean University, Keelung, Taiwan; 5 Institute of Biological Chemistry, Academia Sinica, Taipei, Taiwan; University of Cambridge, UNITED KINGDOM

## Abstract

The genome sequence of *V*. *cholerae* O1 Biovar Eltor strain N16961 has revealed a putative antibiotic resistance (*var*) regulon that is predicted to encode a transcriptional activator (VarR), which is divergently transcribed relative to the putative resistance genes for both a metallo-β-lactamase (VarG) and an antibiotic efflux-pump (VarABCDEF). We sought to test whether these genes could confer antibiotic resistance and are organised as a regulon under the control of VarR. VarG was overexpressed and purified and shown to have β-lactamase activity against penicillins, cephalosporins and carbapenems, having the highest activity against meropenem. The expression of VarABCDEF in the *Escherichia coli* (*ΔacrAB*) strain KAM3 conferred resistance to a range of drugs, but most significant resistance was to the macrolide spiramycin. A gel-shift analysis was used to determine if VarR bound to the promoter regions of the resistance genes. Consistent with the regulation of these resistance genes, VarR binds to three distinct intergenic regions, *varRG*, *varGA* and *varBC* located upstream and adjacent to *varG*, *varA* and *varC*, respectively. VarR can act as a repressor at the *varRG* promoter region; whilst this repression was relieved upon addition of β-lactams, these did not dissociate the VarR/*varRG*-DNA complex, indicating that the de-repression of *var*R by β-lactams is indirect. Considering that the genomic arrangement of VarR-VarG is strikingly similar to that of AmpR-AmpC system, it is possible that *V*. *cholerae* has evolved a system for resistance to the newer β-lactams that would prove more beneficial to the bacterium in light of current selective pressures.

## Introduction

The Gram-negative pathogen *Vibrio cholerae* is the aetiological agent of the acute and potentially fatal diarrhoeal disease, cholera, which is estimated to infect up to 4.3 million people and cause up to 142 000 deaths worldwide yearly [1]. Cholera is contracted when contaminated food or water is ingested [2]. The bacterium then colonizes the small intestine, where a complex regulatory cascade is induced [3], resulting in the production of several important virulence factors, including cholera toxin (CT), an AB-type enterotoxin that is responsible for the secretory diarrhoea that is characteristic of cholera [4].

Treatment of cholera typically entails intensive re-hydration therapy by the intravenous or oral supply of fluids. However, there is a growing concern for the development of antibiotic resistance in *V*. *cholerae* because cholera tends to develop at epidemic levels in developing countries where supplies and assistance is limited. Therefore, the use of antibiotics in these countries is essential to greatly reduce the duration of illness [5], the chances of re-infection [2] and to reduce mortality rates. Moreover, *V*. *cholerae*, which can readily acquire resistance genes, may act as a reservoir for these resistance genes that can be passed onto other pathogenic bacteria by lateral gene transfer [6]. Consequently, it is important to understand the mechanisms of antibiotic resistance in *V*. *cholerae* if we are to develop strategies to circumvent resistance, retaining our capacity to effectively curtail cholera epidemics and the transfer of resistance genes to other pathogenic bacteria.

**Fig 1:**
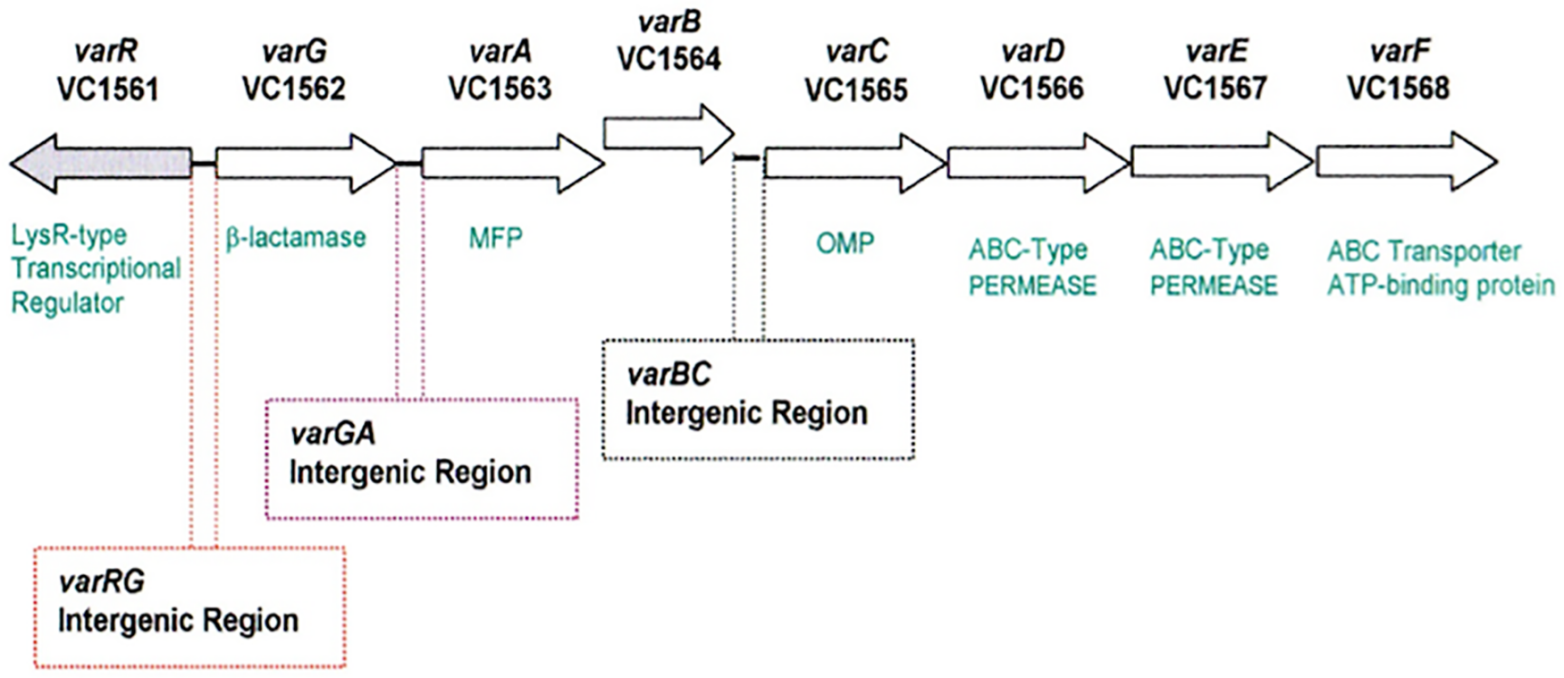
A diagrammatic representation of the *var* operon. The locality of the β-lactamase, *varG*, the MDR *varABCDEF* transporter complex and the divergently transcribed regulatory *varR* genes are shown. Arrows indicate orientation of transcription. Three intergenic regions *varRG*, *varGA* and *varBC* to which VarR is hypothesised to regulate transcription are also illustrated.

An analysis of the genome sequence of *V*. *cholerae* O1 Biovar El Tor strain N16961 revealed a putative antibiotic resistance regulon (which we term *var*, for *V**ibrio* Antibiotic Resistance; Fig 1), apparently consisting of a transcriptional activator (encoded by *varR*), belonging to the LysR family [7], which is divergently transcribed relative to resistance genes for a β-lactamase (encoded by *varG*) and an efflux-pump (encoded by the *varACDEF* genes). A BLAST search revealed that the *var* regulon is not unique to the El Tor strain but is also present in other non-O1 strains. The organisation of the promoters of the *varR* and *varG* genes is similar to that of the well characterised *Enterobacteriacae* β-lactamase *ampR-ampC* regulatory systems of *C*. *freundii* [8–9], *E*. *cloacae* [10] and *P*. *aeruginosa* [11]. An important distinction between the AmpR-AmpC and the VarR-VarG systems is the type of β-lactamase regulated: AmpC belongs to the class of serine-β-lactamases (Sβl, Ambler class C), whereas VarG belongs to the class of metallo-β-lactamases (Mβl, Ambler class B), which target two very different classes of β-lactam antibiotics. Mβls are of fast emerging clinical importance [12–13] owing to their ability to hydrolyse all existing β-lactams, including the newer generation cephalosporins and carbapenems [14]. To exacerbate the situation, the activity of these enzymes cannot be neutralized by current β-lactamase inhibitors and the implementation of such therapeutic inhibitors may take several years [14–16].

That the *var* resistance regulon also incorporates an efflux pump is a novel feature because, although efflux pumps can extrude β-lactams [17–19], this appears to be the first example of both a β-lactamase and an efflux pump occurring in the same regulon. Furthermore, whilst the expression of many antibiotic efflux pumps are regulated by transcriptional regulators, these are generally repressors, which are de-repressed by binding of the same substrates as the pump [20–22]. The genes encoding components of the efflux pump in the *var* regulon are located downstream of VarR, a LysR transcriptional activator (TA). Interestingly, a number of other antibiotic pumps in *V*. *cholerae* have been shown to be regulated by LysR TAs (LTTRs), located upstream of the genes encoding the pumps [23], suggesting that such pumps are regulated by additional factors, possibly secreted by the host.

In general, efflux pumps play an important role in the resistance and virulence of *V*. *cholera*e, since they assist the bacterium in colonizing the small intestine, by providing resistance to toxic antibacterial compounds, such as antimicrobial peptides and bile acids present in the gastrointestinal tract [23–26], and can confer resistance to a range of antibiotics [27–34]. Those belonging to the RND (Resistance-Nodulation-cell Division) superfamily have been studied extensively in *V*. *cholerae* [24,33,34]. There are six RND systems, each of which is separately encoded in an operon structure wherein the RND transporter has at least one associated MFP (membrane fusion protein), whose gene is located upstream of the RND transporter gene. It appears that all six RND efflux systems work with the same OMP (outer-membrane protein), TolC, which is encoded separately on the chromosome [24]. Together these proteins form a tripartite efflux pump that can extrude substrates from the cell. Whilst most of these are involved in conferring resistance, the RND transporters VexH, VexF and VexM appear to have a role in the production of virulence factors, including the cholera toxin and the toxin co-regulated pilus, but this seems to be indirect, possibly by transporting ligands that control expression of these virulence factors [26]. The *var* regulon is also predicted to encode a tripartite efflux pump, but it consists of an inner-membrane ABC (ATP Binding Casssette) transporter, composed of two membrane translocase subunits (VarD and VarE) and an ATPase subunit (VarF), a membrane fusion protein (VarA) and an outer-membrane channel (VarC). The membrane translocase subunits resemble the macrolide transporter MacB and a number of permeases for antimicrobial peptides [35,36]. In *E*. *coli*, MacB, which incorporates both membrane translocase and ABC domains, works with the MFP MacA and the OMP TolC to confer resistance to macrolides [35,36] and is implicated in the secretion of lipopolysaccharides [37], protophorins [38] and protein toxins [39]. MacB has a novel architecture [35,40], consisting of a four transmembrane domain, an N-terminal nucleotide-binding domain (NBD), and a large periplasmic domain, with a structure that resembles that of the periplasmic domain of RND transporters [41]. Whilst MacB forms homodimers [42], MacA has been shown to form hexamers [43] and TolC trimers [44], suggesting that the pump assembles with an IMP:MFP:OMP stoichiometry of 2:6:3. Within the assembled tripartite pump, MacA has been shown to have a functional role in activating MacB [36,42,45,46]. Similarly, RND transporters form trimers [47,48], which are thought to be assembled into a tripartite pump with a stoichiometry of 3:6:3 [49,50].

Herein, we report on the characterization of the *var*-regulon, establishing that VarG is a carbapenemase, that the VarACDEF proteins function as macrolide pump and that the genes encoding these resistance determinants are organised as a regulon under the control of VarR.

## Materials and methods

### Strains and plasmids

*V*. *cholerae* strain CVD101 was used as the source of chromosomal DNA for PCR. The primers used to make constructs and the bacterial strains used to propogate them are given in S1, S2 and S3 Tables, respectively.

#### pSMART-IR_*varRG*_-varG construct

A pSMART-IR_varRG_-varG construct was made to test the β-lactam substrate specificity of VarG. Primers were designed with modified 5’ phosphate ends to enable blunt ended cloning into the pSMART plasmid (Lucigen). The *IR*_*varRG*_*-varG* DNA fragment was amplified using primers var1 and 2. The PCR product was ligated into pSMART and transformed into *E*. *coli* E. cloni (Lucigen) for propagation. The pSMART-IR_varRG_-varG construct was further transformed into *E*. *coli* KAM3 cells [51] for minimal inhibitory concentration (MIC) experiments.

#### pQE100-VarR construct

The *varR* gene was PCR amplified using the primers var3 and var4, which incorporated *BamH*I and *Hind*III restriction sites, enabling unidirectional cloning of the *varR* gene into the pQE-100 vector (Qiagen), to give pQE100-varR, and ensuring expression of VarR was in-frame with the His_6_ tag. pQE100-varR was transformed into *E*. *coli* M15 (pREP4) (Qiagen). A single colony of M15/pREP4/pQE100-varR was used to inoculate 5 ml of 2xYT, supplemented with 25 μg/ml kanamycin (KM) and 100 μg/ml carbenicillin (CB), and cultured overnight. A litre of 2xYT media/ 25 μg/ml KM/ 100 μg/ml CB was inoculated with 3 ml of this overnight culture and grown at 37°C with 180 rpm agitation until an absorbance at 600 nm (A_600_) of 0.5 was reached, then IPTG was added to a final concentration of 0.1mM and growth continued for a further 3.5 hours at 25°C. The culture was harvested by centrifugation (at 6,300 rpm for 8 minutes) and the pellet re-suspended in buffer A (50 ml/ 2 litres of culture) consisting of 20 mM Tris-HCl (pH 8.0), 300 mM NaCl, 10% glycerol (v/v) and 1 mM THP (pre-cooled to 4°C). To a 50 ml cell suspension, 500 units of DNaseI (Sigma) and half a protease inhibitor tablet (Roche) were added. This suspension was passed through a cell disruptor three times at 15 Kpsi and placed on ice for 5 minutes. The cell debris and membranes were separated by ultracentrifugation (at 43K rpm for 1 hour at 4°C). The supernatant was carefully decanted and 300 mM NaCl and 10 mM imidazole (cooled to 4°C) added and rotated for 5 minutes until fully dissolved. One ml of Ni^2+^-agarose (Qiagen) was added to 50 ml supernatant and rotated for 1.5 hours (at 4°C). The Ni^2+^/protein mixture was loaded onto a gravity flow column (BioRad) and the flow through collected. This process was repeated 3 times. The packed column was washed with 20 ml of buffer A supplemented with 50 mM imidazole, and the VarR eluted in 1 ml fractions (up to 8 ml) with buffer A supplemented with 500 mM imidazole. VarR was dialysed against buffer B 20 mM Tris-HCl (pH 8.0), 300 mM NaCl, 10% glycerol, 1 mM THP. VarR was snap frozen and stored at -80°C or used immediately for EMSAs.

#### pET-VarG construct

The *varG* (*VC1562*) gene was PCR amplified using the primers var5 and var6, which incorporated *EcoR*I and *Xho*I restriction sites, respectively, and a sequence encoding an hexahistidine (His_6_) tag downstream. This enabled unidirectional cloning of the *varG* gene into the vector pET-26b(+) (Novagen) to give pET-VarG. pET-VarG was transformed into *E*. *coli* C43(DE3) (Lucigen). The induced cells harboring pET-VarG were collected and re-suspended in 50 mM Tris-HCl (pH 8.0) buffer containing 20% sucrose and EDTA; the cells were pelleted again and re-suspended and incubated in Tris-HCl buffer containing 5 mM MgSO_4_ for 30 min. The recombinant VarG was released into the buffer, and purified and desalted using a Nickel affinity column (Qiagen) and a PD-10 column (Amersham), respectively. VarG was used immediately for β-lactamase assays.

#### pBAD-VarF construct

The *varF* (*VC1568*) gene was PCR amplified using the primers var7 and var8, which incorporated *Nco*I and *Bg*lII restriction sites, respectively, and a sequence encoding an hexahistidine (His_6_) tag. This enabled unidirectional cloning of the *varF* gene into the vector pBAD-B (Life Technologies), to give pBAD-VarF. pBAD-VarF was transformed into *E*. *coli* LMG194 (Life Technologies). An overnight culture was prepared by inoculating 5 ml RM media, supplemented with 100 μg/ml carbenicillin (CB), with a single colony of LMG194/pBAD-VarR. A litre of RM media/ 100 μg/ml CB was inoculated with 3 ml of this overnight culture and grown at 37°C with 180 rpm agitation until an A_600_ of 0.5. At this point IPTG was added to a final concentration of 1 mM and the culture allowed to grow for a further 3.5 hours. The VarF was then purified according to the same protocol used to purify VarR. VarF was desalted using PD-10 columns (Amersham) equilibrated as per manufacturers instruction with buffer B 20 mM Tris-HCl pH 8.25, 50 mM NaCl, 10% glycerol, 1 mM THP. VarF was snap frozen and stored at -80°C or used immediately for further studies.

#### pQE100-VarDEF construct

The *varDEF* (*VC1566-VC1568*) gene cluster was PCR amplified using the primers var9 and var10, which incorporated *Sac*I and *Sma*I restriction sites, respectively, enabling unidirectional cloning of the genes into the vector pQE100 (Novagen), to give pQE100-varDEF. pQE100-varDEF was transformed into *E*. *coli* KAM3 [51] and *(ΔtolC*) TG1 [52].

#### pSYC-VarABCDEF construct

The *vaABCDEF* (*VC1563-VC1568*) gene cluster was PCR amplified using the primers var11 *Nde*I-varABCDEF and var12 *Not*I-varABCDEF, which incorporated *Nde*I and *Not*I restriction sites, respectively, enabling unidirectional cloning of the genes into the vector pSYC, a derivative of pQE100 vector, to give pSYC-varABCDEF. pSYC-varABCDEF was transformed into *E*. *coli* KAM3 [51] and *(ΔtolC*) TG1 [52].

#### Construction of a pSMART-varR-IR_*varRG*_-Cm^R^ vector for MIC experiments

A *varR*-IR_varRG_-*Cm*^*R*^ construct was created, to test whether VarR would bind to the *varR-varG* IR and repress the expression of Cm^R^ that confers resistance to chloramphenicol, in a 2 step cloning process: the *varR*-IR_*varRG*_ part of the construct was amplified using primers var37 and var38, containing the restriction sites *Xba*I and *Nde*I, respectively. Primers were designed with modified 5’ phosphate ends to enable blunt ended cloning into pSMART plasmid. The chloramphenicol resistance gene *Cm*^*R*^ was amplified using primers var39 and var40, containing the restriction sites *Nde*I and *Xho*I, respectively. Both constructs were sub-cloned into pET21a. The IR_v*arRG*_-Cm^R^ construct was amplified using primers var41 and var42, using pET21a- *varR*-IR_v*arRG*_-*Cm*^*R*^ as template DNA, and ligated into pSMART. The varR-IR_v*arRG*_-Cm^R^ construct was amplified using primers var42 and var43, using pET21a-varR-IR_v*arRG*_-Cm^R^ as template DNA, and ligated into pSMART. The constructs were transformed into *E*. *coli* E. cloni (Lucigen) for propagation and then *E*. *coli* KAM3 [51] for MIC experiments.

### Minimal inhibitory concentration (MIC) assays

MICs were performed and assessed by the Microdilution Broth Method established by the National Committee for Clinical Laboratory Standards (NCCLS). In brief, a single colony was used to inoculate 5 ml Mueller Hinton broth (supplemented with appropriate antibiotic) and grown at 37°C with 225 rpm shaking till an absorbance at 625 nm of 0.08–0.1 (0.5 McFarland standard or 1x10^8^ CFU/ml) was reached. The suspension was diluted to 2.5x10^6^ CFU/ml and a measured volume used to inoculate the wells of a 96-well Microtitre plate (final test density of bacteria of 5x10^4^ CFU/well) containing serial dilutions of antibiotics to be tested. Mueller Hinton media was supplemented with 1 mM IPTG if initiation of expression was required. Microtitre plates were incubated statically at 37°C and bacterial growth recorded after 16–20 hours.

### Drug degradation assays

The β-lactamase activity of purified VarG was monitored as the decrease in β-lactam absorbance that results from opening of the β-lactam ring during hydrolysis. The reactions were performed at 30°C in a mixture containing purified VarG, 50 mM Tris-HCl (pH 7.2), 0.1 mg/mL BSA, 100 μM ZnCl_2_ and β-lactam antibiotics, such as ampicillin and imipenem, and the decrease in absorbance monitored. The extinction coefficients and measured wavelengths for the drug degradation test are -820 M^-1^cm^-1^ and 235 nm for ampicillin and piperacillin, -6,500 M^-1^cm^-1^ and 260 nm for cephalothin, -7,600 M^-1^cm^-1^ and 260 nm for cefuroxime, -10,000 M^-1^cm^-1^ and 260 nm for cefepime, -4,000 M^-1^cm^-1^ and 265 nm for moxalactam, 10,940 M^-1^cm^-1^ and 300 nm for meropenem, and -9,000 M^-1^cm^-1^ and 300 nm for imipenem. The rate of β-lactam hydrolysis was measured as a function of the β-lactam concentration and the data fitted to either a hyperbolic or sigmoidal equation using SigmaPlot (Systat Software Inc).

### Size Exclusion Chromatography (SEC)

SEC analyses were performed on an AKTA purifier (Amersham) using Supedex200 PrepGrade HiLoad 16/60 column, equilibrated with 20mM Tris-HCl (pH 8.25), 50mM NaCl and 10% (v/v) glycerol, and run at 1ml/minute. The protein *M*_*r*_ was calculated from a calibration curve constructed using ribonuclease A (15.6KDa), chymotrypsinogen A (22.8KDa), ovalbumin (48.9KDa), albumin (65.4KDa), aldolase (158KDa), catalase (232KDa), ferritin (440KDa) and dextran blue (void volume) standards. The *K*_*av*_ values were calculated using their elution volumes (V_e_), total bed volume (V_t_) and the void volume (V_o_) in the equation *K*_*av*_ = (V_e_-V_o_)/(V_t_-V_o_). A calibration curve was constructed by plotting the log of the *M*_*r*_ of the standards against their *K*_*av*_ values.

### Sedimentation Velocity Analytical Ultra-centrifuge analysis (SV AUC)

Sedimentation velocity (SV) experiments were performed with a Beckman-Coulter XL-A analytical ultracentrifuge (Fullerton, CA, USA). For SV AUC, sample and buffer were loaded into 12-mm standard double-sector Epon charcoal-filled centrepieces and mounted in an An-60 Ti rotor. SV experiments were performed at a rotor speed of 42,000 rpm at 20°C. The sample signal was monitored at 280 nm, and the raw experimental data was analyzed by SEDFIT software (www.analyticalultracentrifugation.com). Plots of *c(s*, *f*_*r*_*) and c(s*, *M)* were generated by MATLAB 7.0 software (MathWork, Inc.). The differential distribution of the sedimentation coefficient and fictional ratio *c*(*s*, *f*_*r*_) was calculated by a *c*(*s*,*) model with Eq 1 [53]:

a(r,t)= ∬c(s,fr)x(s,D(s,fr),r,t)dsdfr (1)

The *c*(*s*, *f*_*r*_) distribution could be transformed to a molar mass distribution for each s-value by a *c(s*, *M)* distribution with Eq 2 [53]:

a(r,t)=∬c(s,M)x(s,D(s,M)r,t)dsdM (2)

### Sedimentation Equilibrium Analytical Ultra-centrifugation analysis (SE AUC)

Sediment-ation equilibrium (SE) experiment were performed with a Beckman-Coulter XL-A analytical ultracentrifuge (Fullerton, CA, USA). For SE AUC, sample and buffer were loaded into 12-mm standard six-sector Epon charcoal-filled centrepieces and mounted in an An-60 Ti rotor. SE experiments were performed at rotor speeds of 16,000, 20,000, 24,000, 28,000, and 32,000 rpm at 20°C. The sample signal was monitored at 280 nm, and experimental data was analyzed by SEDPHAT software (www.analyticalultracentrifugation.com). In SE AUC, the averaged molar mass of a single ideal species is assumed by using the mass conservation model with Eq 3 [54]:

$$A_{r}\;=\;c_{r0}\varepsilon d\text{exp}\left\{M(1-\bar{\nu }\rho \frac{\omega^{2}}{2\text{RT}}\left(r^{2}-r_{0}^{2}\right)\right\}$$ (3)

Where *r* denotes the distance from center of rotation, *r*_0_ is an arbitrary reference radius, ω the angular velocity, *T* is the absolute temperature of the rotor, *R* is the gas constant, $\bar{v}$ is the partial-specific volume, ρ is the solvent density, ε is the extinction coefficient (or analogous signal increment), *d* is the optical pathlength, and *c*_*r*0_ is the concentration at the reference radius.

### Mass Spectrometry Analysis

Intact protein LC-MS analyses were performed on a Waters Acquity nano-UPLC in line with a Waters G2 Q-TOF mass spectrometer. Protein samples (10 μg/mL) were directly infused onto a mass spectrometer through a syringe pump with flow rate 1 μL/min. The G2 Q-TOF mass spectrometer was run in positive ion, high resolution mode with detection in the range of 600 to 2300 m/z. Source parameters were as follows: capillary voltage, 2.50 kV; source temperature, 90°C; desolvation temperature, 200°C; cone gas flow: 20 L/h; the desolvation gas flow, 500 L/h. The protein peak was deconvoluted by the MassLynx MaxEnt1 function according to the following parameters: output resolution, 1.0 Da/channel; output mass range, 35–85 KDa; uniform Gaussian width at half height, 0.75 Da; minimum intensity ratios, 30% for left and right; iteration to convergence for completion.

### Dye-accumulation assay

One colony of *E*. *coli* KAM3, transformed with pQE100, pQE100-varDEF or pSYC-varABCDEF, was inoculated into Mueller Hinton broth and grown over-night (for approx. 8 to 10 h). The cells were collected by centrifugation (at 6,300 rpm for 8 minutes) and washed twice and finally re-suspended with PBS buffer (adjusting the cell density in PBS to an A_600_ of 0.6). The *E*. *coli* cells (150 μl in PBS) were placed into the well of a 96-well plate (in triplicate), D-glucose was added to a final concentration of 25 mM and left to incubate for 3 min, after which time 1 μM Hoechst 33342 (Sigma) was added and the fluorescence of the cells monitored with time in a (BioTek) fluorescence plate reader (Excitation 360 nm, Emission 460 nm).

### ATPase activity measurements

The ATPase activity of VarF was determined using a malachite green assay to monitor Pi production [55]. The reaction mixture containing 20 mM Tris (pH 7.5), 200 mM NaCl, 10 mM MgCl_2_ and 0.5 μM VarF, which was mixed with varying concentrations of adenosine 5’-triphosphate disodium salt (ATP), and incubated at 37°C. The ATPase reaction was initiated by adding 5 mM MgCl_2_, and sample were removed from the reaction mixture, at 1 minute intervals over 10 mininutes, and mixed with 0.5 M EDTA to stop the reaction. Malachite green solution (one vol of 4.2% (w/v) ammonium molybdate in 4 M HCl mixed with three vols of 0.045% (w/v) malachite green) was added to each sample and the 610 nm absorbance measured. The rate of ATP hydrolysis was measured as a function of the ATP concentration and the data fitted to a sigmoidal equation using SigmaPlot (Systat Software Inc).

### Electrophoretic mobility shift assays (EMSAs)

Gel-shift assays were used to detect the binding of VarR to the intergenic regions (IRs) apparent within the *var* gene cluster and oligonucleotides derived from the sequences of IRs. Typically, 100 ng of purified VarR was incubated on ice for ten minutes with 1 ng of labelled PCR fragment or oligonucleotide in 50 mM Tris–HCl (pH 7.5), 5 mM EDTA, 1 mM DTT, 100 μgml^-1^ BSA, 5% glycerol. Samples were applied to a low ionic strength 4% polyacrylamide gel and electrophoresed for 1.5 hours at 190 V. The gel was dried and autoradiographic profiles were produced. Specificity of binding was assayed by incubating VarR with 1 ng of labelled oligonucleotide and 1 ng (and increasing quantities) of unlabeled oligonucleotide to give the duplex probe. VarR was also incubated with drugs to test their modulatory effect upon binding to target DNA. Approximate DNA concentrations of oligonucleotides were calculated by liquid scintillation. DNA fragments spanning the different IRs were PCR amplified as detailed below:

#### *varR-varG* IR

A 302 bp DNA fragment was amplified using primers var13 and 14. This DNA fragment incorporated 120 bp from the 5’ ends of the *varR* (transcription factor) and *varG* (β-lactamase) genes, encasing a 63 bp IR hypothesised to contain the promoters of these respective genes. A 151bp DNA fragment was amplified using primers var13 and 15. This DNA fragment incorporated 120 bp from the 5’ end of the varR and the first 31 bp of the 63 bp IR. A 151 bp DNA fragment was amplified using primers var14 and 16. This DNA fragment incorporated 120 bp from the 5’ end of the *varG* (β-lactamase) gene and the last 31 bp of the 63 bp IR. The duplex DNA fragments were labelled with γ-^32^P (ATP) (Amersham) using T4 polynucleotide kinase (Fermentas) and unincorporated nucleotides were removed using Micro Bio-Spin columns (Bio-Rad). Labelled duplex-DNA was purified by electrophoresis at 190 V for 2.25 hours (Protean II, Bio-Rad) on a 10% (w/v) TBE polyacrylamide gel. A 31 bp oligonucleotide incorporating the first half of the 63 bp IR was produced by labelling single stranded oligonucleotide varR17 and then annealing (95°C for 2 minutes) with three-fold of its complimentary strand varR18. The annealed duplex DNA was allowed to cool, unaided, to ambient temperature. A 32 bp oligonucleotide containing the second half of the 63 bp IR was produced as described above using oligonucleotides, var19 and var20 respectively.

#### *varG-varA* IR

A 415 bp DNA fragment was amplified using primers var21 and 22. This DNA fragment incorporated 120 bp from the 5’ ends of the *varG* (β-lactamase) and *varA* (MFP) genes, encasing a 176 bp IR hypothesised to contain the promoters of the latter gene. A 207 bp DNA fragment was amplified using primers var21 and 23. This DNA fragment incorporated 120 bp from the 5’ end of the *varR* gene and the first 87 bp of the 176 bp IR. A 208bp DNA fragment was amplified using primers var22 and 24. This DNA fragment incorporated 120 bp from the 5’ end of the *varA* gene and the last 88 bp of the 176 bp IR. A 176 bp DNA fragment was amplified using primers var25 and 26. This DNA fragment encased a 176 bp IR hypothesised to contain the promoter of the *varA* gene. An 88 bp DNA fragment was amplified using primers var25 and 27. This DNA fragment incorporated the first 88 bp of the 176 bp IR. An 88 bp DNA fragment was amplified using primers var26 and 28. This DNA fragment incorporated the last 88 bp of the 176 bp IR. A 30 bp oligonucleotide containing the first 12 bp of the 176 bp IR was produced using primers var29 and 30. A series of eight 30 bp oligonucleotides (varGA1 –varGA8) spanning the *varGA* IR were used as probes for VarR binding.

#### *varB-varC* IR

A 194 bp DNA fragment was amplified using primers var31 and 32. This DNA fragment incorporated 50 bp from the 5’ end of the *varB* and 120 bp of the 5’ end of the *varC* genes, encasing a 25 bp IR hypothesised to contain the promoter of the downstream genes. A 97 bp DNA fragment was amplified using primers var31 and var33. This DNA fragment incorporated 50 bp from the 3’ end of the *varB* gene, the 25 bp IR and 22 bp from the 5’ end of the *varC* gene. A 97 bp DNA fragment was amplified using primers var32 and var34. This DNA fragment incorporated 97 bp from the 5’ end of the *varC* gene. A 25 bp oligonucleotide containing the *varB-varC* IR was produced using primers var35 and 36.

A positive control was run using the transcriptional regulator MtrR binding to a 30 bp oligonucleotide (VarPC) corresponding to the *mtr* promoter [56], whilst a 30 bp non-specific oligonucleotide (VarNC), which did not bind VarR, was run as a negative control. In negative control titrations, the VarNC probe was used at concentrations upto three-orders of magnitude that of the specific labelled-probe: in all cases the VarNC probe, of unrelated sequence, failed to displace the labelled-probe, indicating that the interactions were specific.

### Ethics statement

This project did not include any work with live animals for which ethical approval is required.

## Results

### Identification of a novel antibiotic resistance operon in *Vibrio cholerae*

The database of the National Center for Biotechnology Information (NCBI) was used to examine the chromosomes of *V*. *cholerae* El Tor O1 Biovar Eltor strain N16961 in which the genome has been sequenced [57]. A set of putative structural genes (open reading frames (ORFs) *VC1562-VC1568*) were identified on chromosome I of the strain N16961, which were indicative of a resistance regulon, consisting of a metallo-β-lactamase (Mβl) VarG (ORF *VC1562*) and a multi-component tripartite ABC transport system VarACDEF (ORFs *VC1563*, *VC1565-VC1568*). The latter efflux-pump, which resembles those involved in the transport of macrolides and antimicrobial peptides in other bacteria, consists of an inner-membrane ABC-transporter, composed of two membrane translocase subunits (VarD and VarE) and an ATPase subunit (VarF), a membrane fusion protein (VarA) and an outer-membrane channel (VarC). Directly upstream of the regulon is the gene for a transcription factor VarR (VC1561) that belongs to the LysR transcriptional regulatory protein (LTTR) family, which is divergently transcribed relative to the putative resistance genes. The position of this regulatory protein suggests that it may co-regulate the expression of these two distinct resistance mechanisms. There are three intergenic regions within the *var* operon in which VarR could interact to regulate transcription, designated *varRG*, *varGA* and *varBC* in Fig 1. We sought to test whether these genes could confer antibiotic resistance and are organised as a regulon under the control of VarR.

### VarG is a metallo-β-lactamase

A BLAST search and subsequent sequence alignment of the protein VarG (encoded by ORF *VC1562*) indicated that it encodes an Mβl (Ambler class B) metallo-β-lactamase (GenBank accession number AAF94716). Chromosome one of *V*. *cholerae* 01 Biovar Eltor strain N16961 was analysed for additional chromosomally encoded β-lactamases, however VarG was the only β-lactamase to be found.

Although members of the Mβl family exhibit considerable sequence diversity, they do share some degree of conserved sequences within the active sites of the enzyme [58]. The conserved nature of the active sites in Mβls is such that the architecture can be virtually superimposable on one another. Conserved residues are involved in forming interactions with one (monozinc-Mβls) or two (bizinc-Mβls) Zn^2+^ ions; and exhibit a hallmark consensus sequence for two active sites (Zn1 and Zn2) of HXHXD(X)_a_H(X)_b_C(X)_c_H, where X indicates any amino acid, a = 55–74, b = 18–24 and c = 37–41 intervening residues, respectively [58]. An amino acid alignment of VarG with the sequences of other Mβls identified residues that have the same consensus sequence: His152, His154, Asp156, His238, Asp257 and His301. In VarG, this zinc-binding motif is hypothesized to form two metal-binding sites, with residues His152, His154 and His238 forming the Zn1 site and residues Asp156, Asp257 and His301 form the Zn2 site. The absence of the single conserved cysteine in the consensus sequence of VarG (C257D) may affect binding affinity for Zn^2+^ compared to other Mβls with the complete motif [59]. However, mutational analysis has suggested the irrelevance of this cysteine for binding and hydrolysis in the bi-zinc enzyme as the respective cysteine was substituted and still demonstrated the ability of the derivative to bind two Zn^2+^ ions [60,61].

According to the Ambler classification Mβls are further classified into three subgroups, B1, B2 and B3. Subgroups B1 and B2 are generally grouped together due to shared sequence homology. Subgroup B3 on the other hand is grouped separately from B1 and B2 due to minimal sequence homology. However, substitutions in the conserved residues of the Zn^2+^-binding sites have enabled some distinction between the three subclasses. In Zn1, the standard consensus sequence involving three histidines can be found in subclasses B1 and B3, but a histidine residue is replaced by an asparagine in subclass B2. In Zn2, the cysteine in the aspartic acid-cysteine-histidine triad is substituted by a histidine in the subclass B3 [61]. Analysis of the conserved zinc-binding motif of VarG did not verify which subclass it belongs to, since it does not show any of these substitutions. However, Mβls can also be sub-grouped on the basis of their substrate specificity: the subclass B1 enzymes prefer penicillins and cephalosporins as substrates, whilst the subclass B2 enzymes prefer carbapenems as substrates and the subclass B3 enzymes prefer penicillins as substrates [62].

The specificity of the VarG β-lactamase was assessed by expressing VarG, using the vector pSMART-IR_varRG_-varG, in *E*. *coli* KAM3 cells [51], which are hypersensitive to a range of antibiotics, and determining the MIC values for a range of β-lactams (Table 1). Expressing *varG* conferred a modest resistance to penicillins and cephalosporins and a relatively high resistance to carbapenems on the KAM3 cells (Table 1). Although VarG is predicted (using Signal BLAST) to possess a signal-peptide, the level of resistance would reflect the amount of VarG reaching the periplasm, which may be less than optimal in *E*. *coli*.

**Table 1:** MIC values for *E*. *coli* KAM3 cells expressing VarG.

β-lactam	IC_50_ (μg/mL)		Relative resistance^a^
	KAM3/pSMART (Control)	KAM3/pSMART-IR_*varRG*_*-VarG*	
Penicillin G	4	16	4
Ampicillin	0.5	2	4
Carbenicillin	0.5	2	4
Nafcillin	1	4	4
Cephalothin	1	4	8
Cefepime	0.25	2	8
Imipenem	0.125	8	64
Meropenem	0.025	8	320

^a^Relative resistance is the ratio of the IC_50_ for KAM3/pSMART-IR_*varRG*_*-VarG* to the IC_50_ for KAM3/pSMART

**Fig 2:**
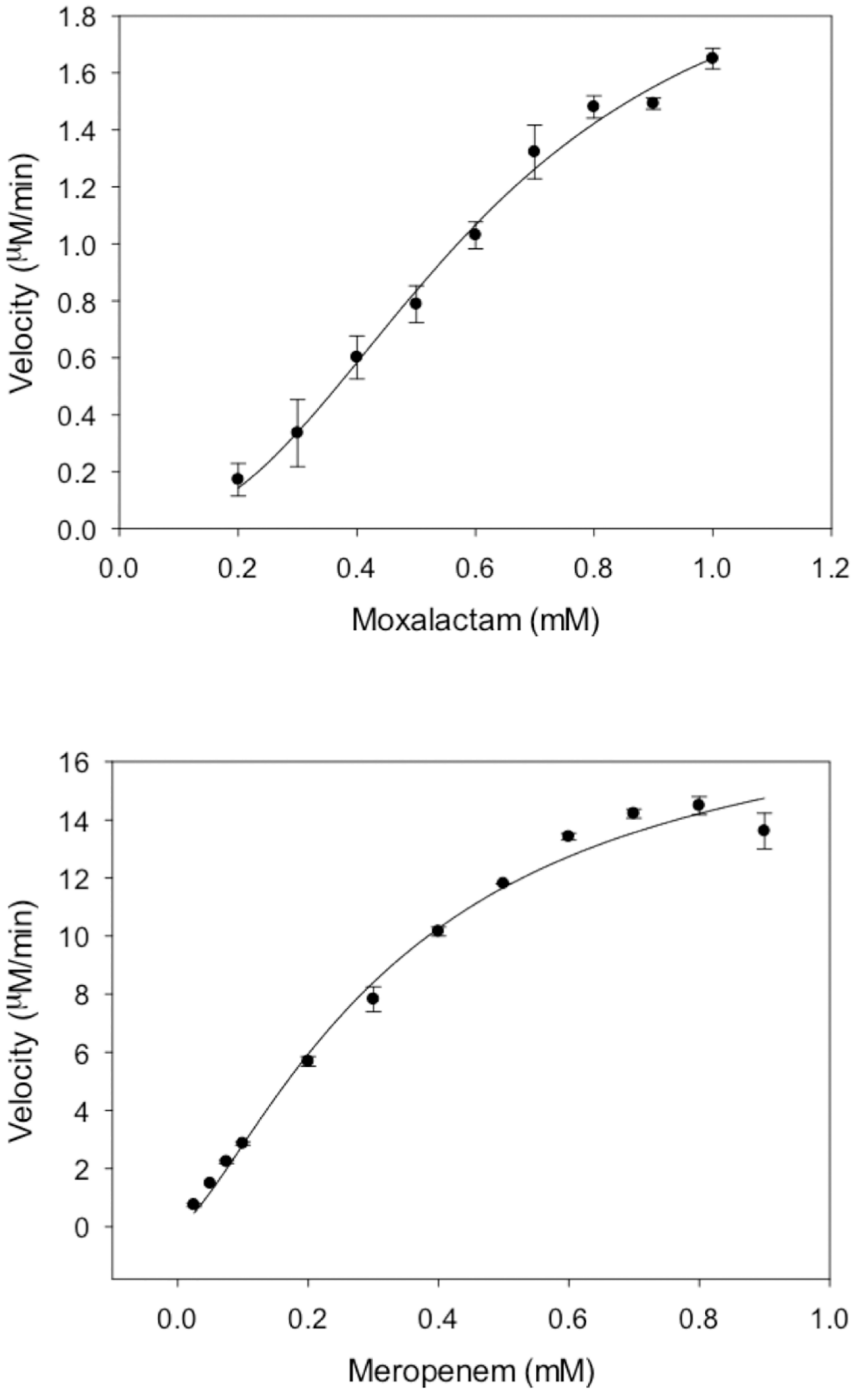
The steady-state kinetics of β-lactam degradation by the VarG β-lactamase. The β-lactamase activity of purified VarG was monitored as the decrease in β-lactam absorbance that resulted from opening of the β-lactam ring during hydrolysis. The rate of (A) moxalactam and (B) meropenem β-lactam hydrolysis was measured as a function of the β-lactam concentration and the data fitted to a sigmoidal equation. The data indicated that the β-lactams moxalactam and meropenem are hydrolysed by VarG with values for the V_max_, K_m_ and Hill Coefficient of 2.1 (± 0.22) and 18.6 (+ 1.92) μMmin^-1^, 0.6 (± 0.06) and 0.35 (± 0.063) mM, and 2.4 (± 0.29) and 1.4 (± 0.17), respectively.

Inorder to further characterize VarG, it was overexpressed, using a pET vector, and purified as a His-tagged protein from *E*. *coli* C43 (DE3). Initially, using pET21a to overexpress VarG, we found that VarG released from the cytoplasm had little activity, and so subsequent we used pET26b, to fuse the *pelB* sequence to *varG*, to better target it to the periplasm. VarG in the periplasm was released from the cells by osmotic shock. The identity of the purified protein was confirmed by ESI-Q-TOF MS/MS protein sequencing; whilst size-exclusion chromatography, analytical ultra-centrifugation and MS/MS mass-spectrometry all indicated that VarG forms a dimer (S1 Fig). The ability of VarG to act as a β-lactamase was determined from drug degradation assays, in which the hydrolysis of β-lactam antibiotics, due to opening of the β-lactam ring, is followed as a decrease in absorbance. In assays were (100 μM) ZnCl_2_ was omitted from the assay buffer and (1 mM) EDTA was added, no activity was noted, indicating VarG had a requirement for Zn^2+^ ions. Typically, a plot of the initial rate of β-lactam hydrolysis as a function of the β-lactam concentration was sigmoidal, indicating co-operativity between the subunits of dimeric VarG. As exemplified by the data for moxalactam and meropenem in Fig 2, the data was best fitted by non-linear regression to curves with Hill coefficients of 2.4 ± 0.29 and 1.4 ± 0.17, respectively. This behavior is consistent with positive co-operativity between the binding sites of dimeric VarG. The k_cat_ and K_m_ values for a range of substrates were determined (Table 2) indicating that VarG showed relatively high activity on carbapenems (i.e. meropenem *k*_*cat*_*/K*_*m*_ = 2.6 x 10^5^ M^-1^s^-1^) and moderate activity on penicillins (i.e. ampicillin *k*_*cat*_*/K*_*m*_ = 1.8 x 10^3^ M^-1^s^-1^) and cephalosporins (i.e. cefepime *k*_*cat*_*/K*_*m*_ = 1.9 x 10^2^ M^-1^s^-1^), and no activity on monobactams (i.e. Aztreonam). This difference in catalytic activity did not arise from a significant difference in the affinity for the different substrate, for which there was only a 6-fold increase between the best substrate (e.g. meropenem K_m_ = 0.34 mM) and the worst (e.g. imipenem, K_m_ = 1.7 mM). This data is consistent with VarG being catergorized as an Ambler class B2 Mβl, which have a preference for carbapenem substrates [62].

**Table 2:** Steady state kinetic parameters for VarG hydrolysis of β-lactam antibiotics.

β-lactams	Conc range (μM)	*V*_*max*_ (μM/min)	*K*_*m*_ (mM)	*k*_*cat*_ (s^-1^)	*k*_*cat*_ / *K*_*m*_ (M^-1^s^-1^)	*Hill Coeff*. *n*
Ampicillin	250–2500	28.9±1.64	1.00±0.08	2.41	2.4 x 10^3^	1.73
Piperacillin	100–1000	1.98±0.26	0.48±0.07	0.11	2.3 x 10^2^	2.07
Cephalothin	50–800	0.76±0.09	0.33±0.05	0.06	1.8 x 10^2^	1.95
Cefuroxime	50–1200	1.74±0.31	0.63±0.18	0.15	2.4 x 10^2^	1.45
Cefepime	100–1200	2.51±1.63	1.11±0.81	0.21	1.9 x 10^2^	1.75
Moxalactam	200–1200	1.83±0.09	0.52±0.03	0.15	2.9 x 10^2^	2.40
Imipenem	250–2500	70.0±22.91	1.68±0.79	233.6	1.4 x 10^5^	1.48
Meropenem	25–1000	18.64±1.92	0.34±0.06	62.13	1.8 x 10^5^	1.39
Aztreonam	50–1500	ND	ND	ND	ND	ND

ND—not determined

In terms of its activity, VarG has about an order of magnitude lower catalytic activity with meropenem (i.e. 1.8 x 10^5^ M^-1^s^-1^) than NDM-1 (i.e. 2.6 x 10^6^ M^-1^s^-1^) and ImiS (i.e. 2.4 x 10^6^ M^-1^s^-1^), but nearly equivalent to that of CphA (2.1 x 10^5^ M^-1^s^-1^) [61,62]. VarG had an affinity for meropenem (i.e. 340 μM) that was lower than that of NDM-1 (i.e. 54 μM) but similar to those of two Ambler class B2 enzymes, ImiS (i.e. 250 μM) and CphA (i.e. 308 μM). Although VarG exhibited the highest catalytic activity (i.e. k_cat_/K_m_) for meropenem, it had the highest k_cat_ for imipenem (i.e. 233 s^-1^), but this was offset by a lower affinity/higher K_m_ (i.e. 1.7 mM) in the catalytic activity (i.e. 1.4 x 10^5^ M^-1^s^-1^). NDM-1 displays similar behavior, in that it has the highest k_cat_ for penicillin (i.e. 720 s^-1^, compared to 195 s^-1^ for imipenem) but this is offset by a lower affinity (i.e. 240 μM) [63,64].

### VarDEF is an ABC-transporter of antibiotics

An analysis of the secondary structures of VarD and VarE (encoded by the ORFs *VC1566* and *VC1567*) indicated that they are integral membrane proteins, each composed of a 4-helix transmembrane segment and a large periplasmic domain. A BLAST search and subsequent sequence alignment of the proteins revealed that they have homology with a number of antimicrobial peptide permeases and with the macrolide transporter MacB, which is also predicted to have a similar topology but with an additional N-terminal nucleotide-binding domain (NBD) [35,40]. An analysis of the secondary structure of VarF (encoded by the ORF *VC1568*) indicated that it is an ABC protein, presumably serving a similar function to the NBD in MacB. Whilst most ABC exporters tend to have an architecture in which the transmembrane- and nucleotide-binding domains/subunits have fused into a single protein [65], there are a few examples of those that use separate subunits (e.g. the *Streptomyces peucetius* DrrAB pump [66]). In order to characterize VarF, it was overexpressed and purified as a His-tagged protein from *E*. *coli* LMG194/pBAD-B-*varF*. VarF was shown to have ATPase activity, using a malachite green assay, which increased sigmoidally with the ATP concentration (S2 Fig), indicating a K_m_, Hill Coefficient and V_max_ of 795 (± 81.2) μM, 1.87 (± 0.29) and 34.6 (± 2.3) nmoles Pi/min/mg, respectively. Consistent with a SEC analysis, which revealed a significant population of VarF dimers (S1 Fig), this data indicated that VarF forms a functional dimer in which there is positive co-operativity between the nucleotide binding sites. In comparison, MacB was characterized by a K_m_ and V_max_ of 374 μM and 8.9 nmol Pi/min/mg [42], respectively. Although, co-operativity between the nucleotide binding sites of MacB was not detected, such behavior has been reported for a number of ABC transporters [67]. Interestingly though, our studies reveal that such co-operativity can exist between the soluble ATPase subunits of an ABC exporter.

To determine if VarDEF form a functional pump for antibiotics, the proteins were expressed from *E*. *coli* KAM3/pQE100-VarDEF. The resistance to a range of antibiotics was determined for the KAM3 strain [51], which is hypersensitive to antibiotics due to deletion of *acrB*, which encodes the inner-membrane RND transporter that is part of the AcrABTolC tripartite multidrug pump. This established that the proteins conferred resistance to a range of antibiotics, but most noticeably to macrolides. The MIC for spiramycin was 8-fold higher for cells expressing the VarDEF pump than for control cells without the pump (but transformed with the empty plasmid) (Table 3).

**Table 3:** MIC values for *E*. *coli* KAM3 cells expressing VarDEF.

Drug group and drug	IC_50_ (μg/mL)		Relative resistance^a^
	KAM3/pQE100 (Control)	KAM3/pQE100-*varDEF*	
**Macrolide**			
Azithromycin	1	4	4
Clarithromycin	2	8	4
Erythromycin	4	32	8
Spiramycin	17.25	138	8
**Glycopeptide**			
Vancomycin	32	32	1
**Nitrofuran**			
Furazolidone	21	42	2
**Tetracycline**			
Doxycycline	4	8	2
Tetracycline	0.66	1.32	2
**Quinolone**			
Ciprofloxacin	0.003	0.006	2
Norfloxacin	0.007	0.015	2
Ofloxacin	0.06	0.125	2
**Sulfonamide**			
Co-trimoxazole	31.75	63.5	2
**Aminoglycoside**			
Kanamycin	16	32	2
Streptomycin	101.56	203.12	2
**Inhibitors**			
Reserpine	18.75	18.75	1
**Others**			
Chloramphenicol	56.93	14.23	0.25
Rifampicin	16.25	16.25	1
Hochest 33342	2	4	2

^a^Relative resistance is the ratio of the IC_50_ for KAM3/pQE100-varF to the IC_50_ for KAM3/pQE100

N.D.: not determined

### VarDEF functions as part of a tripartite pump

The *varDEF* genes are found adjacent to the *varA*, *varB* and *varC* genes. A BLAST search and subsequent sequence alignment of the proteins encoded by *varA* and *varC* indicated that these genes encode a membrane fusion protein (MFP), with a single N-terminal *α*-helix, and an outer membrane protein (OMP) channel. Interestingly, *varB* appears to encode a 40 amino acid remnant of the C-terminal domain of an MFP.

**Fig 3:**
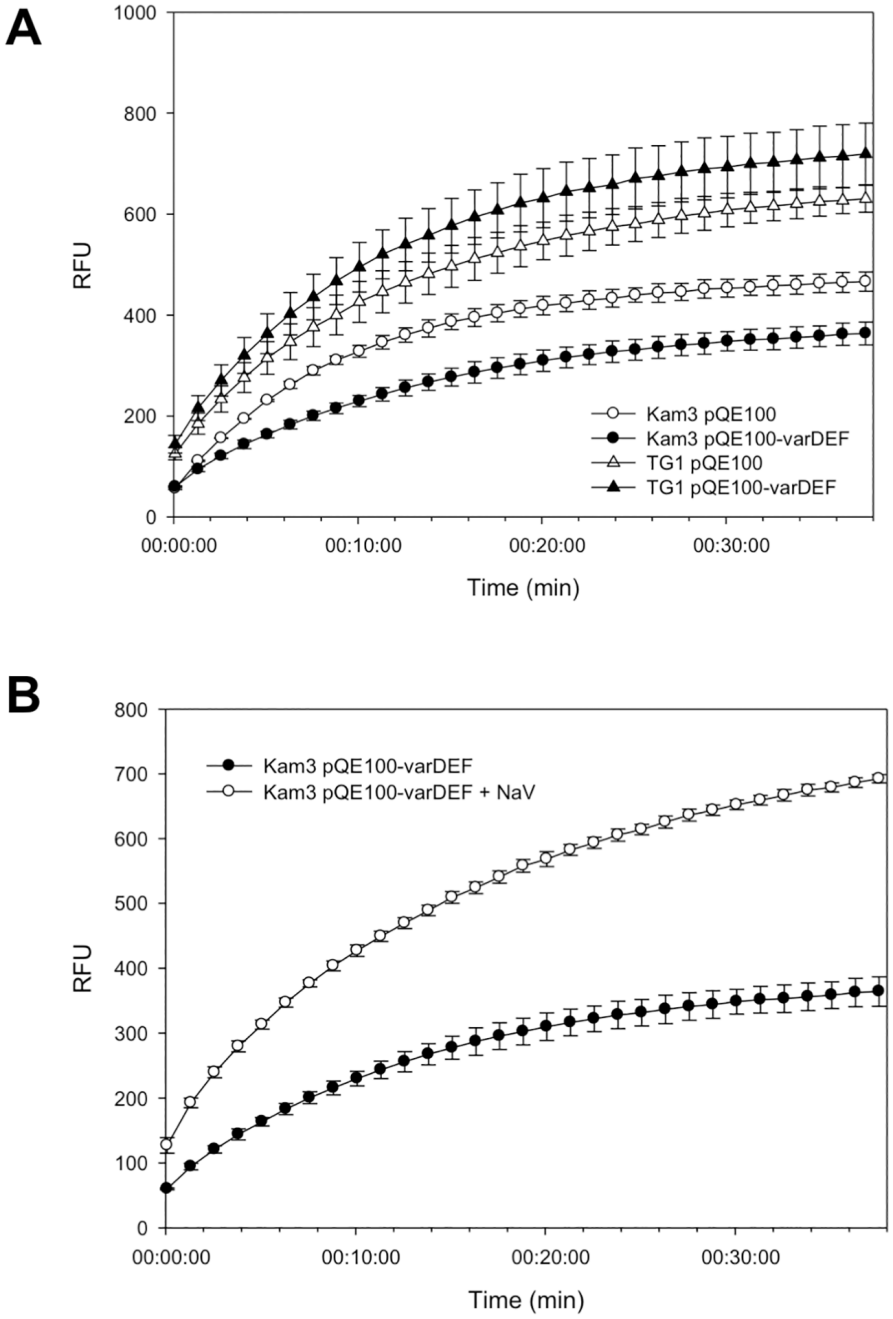
Accumulation of Hoechst 33342 by *E*. *coli* KAM3 and *E*. *coli* TG1 cells harboring pQE100-*varDEF*. **(A)** The *E*. *coli* cells (150 μl in PBS) were placed into the well of a 96-well plate, D-glucose was added to a final concentration of 25 mM and left to incubate for 3 min, after which 2.5 μM Hoechst 33342 (Sigma) was added and the fluorescence of the cells monitored with time in a (BioTek) fluorescence plate reader (Excitation 360 nm, Emission 460 nm). **(B)** Effect of the ATPase inhibitor sodium orthovanadate (NaV) on the accumulation of Hoechst 33342 (2.5 μM) by *E*. *coli* KAM3 harboring pQE100-*varDEF*. Hoechst 33342 was incubated for 38 min with *E*. *coli* KAM3 harboring pQE100-*varDEF* and 40 μg/ml NaV. Cells treated with NaV accumulated substantially more Hoechst 33342 consistent with inactivation of the VarDEF ATP-driven efflux pump. All assays were performed in triplicate.

Since VarDEF conferred resistance to macrolides in the *E*. *coli* (*ΔacrB*) KAM3 strain, we tested whether it would confer resistance in the *E*. *coli* (*ΔtolC*) TG1 strain [52]: VarDEF did not confer resistance to antibiotics when expressed in *(ΔtolC*) TG1 (S4 Table), suggesting that VarDEF interacts with TolC in KAM3 cells. To further test this hypothesis we monitored the uptake of the fluorescent dye Hoechst 33342 by both KAM3 and (*ΔtolC*) TG1 cells. The dye was accumulated to lower levels in KAM3 cells expressing VarDEF than by control cells without the pump (Fig 3A), consistent with extrusion of the dye from the cells by the VarDEF pump. Furthermore, when the cells expressing VarDEF were treated with the ATPase inhibitor sodium orthovanadate (NaV) they accumulated more dye, consistent with VarDEF acting as an ABC exporter of the dye (Fig 3B). In contrast, the (*ΔtolC*) TG1 cells accumulated much higher levels of the dye than KAM3 cells, with (*ΔtolC*) TG1 cells expressing *varDEF* accumulating the dye to the highest level, indicating that the VarDEF pump was unable to remove the dye from the (*ΔtolC*) TG1 cells and might be acting as a leakage pathway for entry of the dye into the cells (Fig 3A).

**Fig 4:**
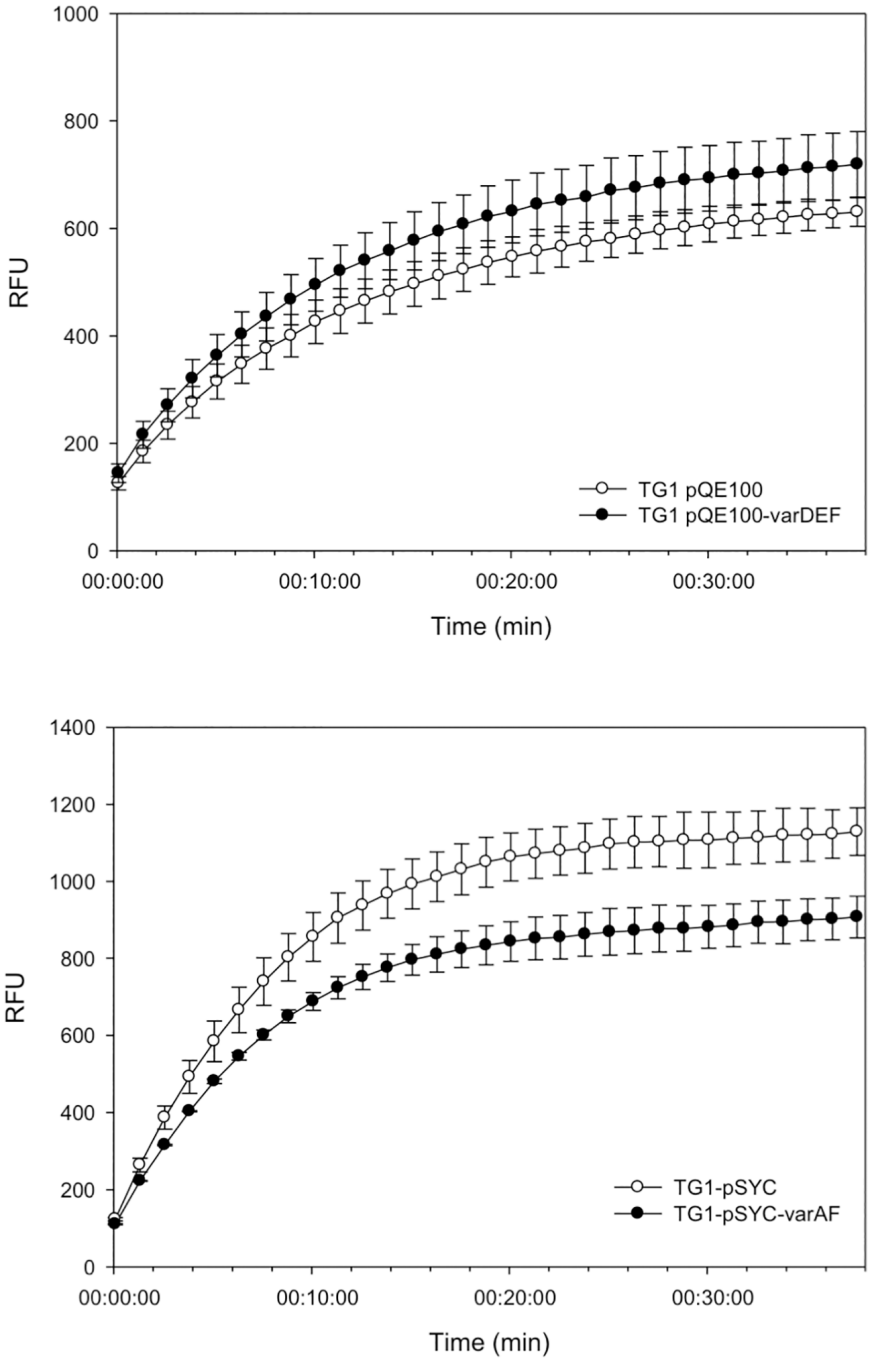
Accumulation of Hoechst 33342 (2.5 μM) by *E*. *coli* TG1 cells harboring pQE100-VarDEF and pSYC-VarABCDEF. The *E*. *coli* cells (150 μl in PBS) were placed into the well of a 96-well plate, D-glucose was added to a final concentration of 25 mM and left to incubate for 3 min, after which 2.5 μM Hoechst 33342 (Sigma) was added and the fluorescence of the cells monitored with time in a (BioTek) fluorescence plate reader (Excitation 360 nm, Emission 460 nm). Assays were performed in triplicate.

Subsequently, we found that *E*. *coli* KAM3/pQE100-varABCDEF cells, expressing *varABCDEF*, conferred a significantly higher resistance to macrolides than KAM3 cells expressing *varDEF* (Table 4). The MIC for spiramycin was 32-fold higher for cells expressing the VarABCDEF pump than for control cells without the pump (but transformed with the empty plasmid). Since we had previously found that in contrast to *E*. *coli* KAM3 cells, *E*. *coli* (*ΔtolC*) TG1 cells expressing VarDEF could not confer resistance to macrolides and accumulated more Hoechst 33342, we tested whether (*ΔtolC*) TG1 cells expressing *varABCDEF* would accumulate less Hoechst 33342. As shown in Fig 4, *E*. *coli* (*ΔtolC*) TG1/pQE-varDEF cells accumulated more Hoechst 33342 than (*ΔtolC*) TG1/pQE100 cells, whilst (*ΔtolC*) TG1/pSYC-varABCDEF cells accumulated less Hoechst 33342 than (*ΔtolC*) TG1/pSYC cells. We conclude that the VarDEF ABC transporter works as part of a tripartite drug pump in conjunction with the MFP VarA and the OMP VarC, which increase the efficiency of drug export from the cell. Considering that such a tripartite pump would have the capacity to confer resistance to antibiotics, such as β-lactams, that target the periplasm, we tested but found that the pump did not confer increased resistance to meropenem (Table 3).

**Table 4:** MIC values for *E*. *coli* KAM3 cells expressing VarABCDEF.

Drug group and drug	IC_50_ (μg/ml)		^a^Relative resistance
	KAM3/pSYC (control)	KAM3/pSYC-*varABCDEF*	
**Macrolide**			
Azithromycin	1	16	16
Clarithromycin	2	16	8
Erythromycin	4	32	8
Spiramycin	8.625	276	32
**β-lactam**			
Meropenem	1	1	1

^a^Relative resistance is the ratio of the IC50 for Kam3/pSYC-*varABCDEF* to the IC_50_ for Kam3/pSYC

### VarR functions as a transcriptional regulator of the *var* operon

#### VarR binds to the *varRG* intergenic region

**Fig 5:**
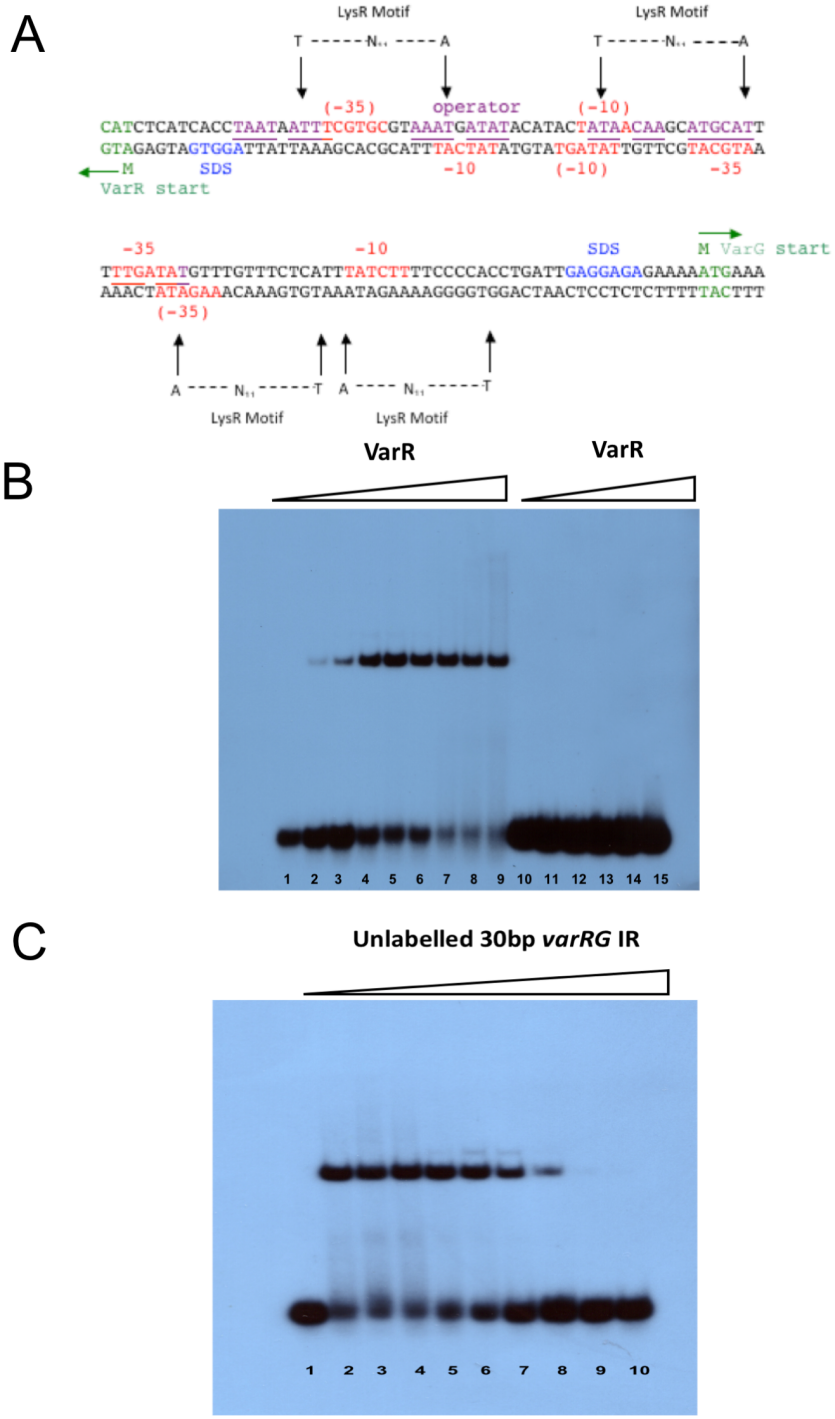
VarR binds to the varR-varC intergenic region. **(A)** The nucleotide sequence of the 111bp *varRG* intergenic region containing the putative promoter sequences represented by -35 and -10 regions (highlighted red). The Shine-Dalgarno sequence is highlighted blue. The operator site which is hypothesised to act as the binding site for VarR is highlighted purple and/or underlined if overlapping with promoter sites. The start codons and the deduced amino acid for VarR and VarG are highlighted in green. **(B)** EMSA using increasing titrations of VarR with 30 bp putative operator *varR-varG* IR, including 30 bp non-specific DNA Lanes 1 to 9. Titrations of VarR (0, 1.25, 2.5, 5, 10, 25, 50, 100, 200ng, respectively) with 0.08ng 30 bp *varR-varG* IR. Lanes 10 to 15, titrations of VarR (0, 5, 10, 50, 100, 200ng, respectively) with 0.08ng 30 bp non-specific DNA. **(C)** EMSA of 50 ng VarR/ 0.08ng 30 bp *varR-varG* IR DNA (labelled) complex with titrations of unlabelled 30 bp *varR-varG* IR DNA. Lane 1, 0.08ng 30 bp *varR-varG* IR DNA only. Lanes 2 to 10, competition assay of 50 ng VarR/ 0.08 ng 30 bp *varR-varG* IR DNA (labelled) complex with titrations of unlabelled 30 bp *varR-varG* IR DNA (0, 0.125, 0.25, 0.5, 1, 2, 5, 10, 20ng, respectively).

The intergenic region (IR) between the *varR* and *varG* genes was analysed (Fig 5A). Identification of the initiation codons for the *varR* and *varG* ORFs indicated a 111 bp IR that accommodates two putative overlapping promoters, one for *varR* and the other for *varG*, therefore VarR has the potential to control transcription in a bidirectional manner. Within the 111 bp *varRG* IR, putative -10 and -35 sites and two T-N_11_-A motifs were identified for each of the *varR* and *varG* promoters. Upstream from the initiation codon of *varG*, a likely bacterial promoter was found with a -35 region (i.e. TTGATA) and a -10 region (i.e. TATCTT) separated by 15 bp. Divergent to the *varG* ORF, a putative promoter was found upstream of the initiation codon of *varR* containing a -35 region (i.e. TACGTA) and a -10 region (i.e. TCGTGC) separated by 17 bp. The base pair separation between these putative promoter regions has been demonstrated to be optimal for promoter strength [68]. Within the *varRG* IR, a likely -65 LTTR recognition-binding site, incorporating a T-N_11_-A motif, was identified for *varG*, which overlaps the -35 site for *varR*. Moreover, this site sits within a putative operator site, identified by the presence of partial inverted repeat sequences, which is hypothesized to be the repressor binding site for VarR; binding at this site may prevent transcription of *varG* and its own expression through autoregulation.

To test whether VarR was able to bind to these sites, it was overexpressed and purified as a His-tagged protein, from *E*. *coli* M15 (pREP4)/pQE-100-varR, and used in electrophoretic mobility shift assays (EMSAs) with DNA fragments corresponding to the sequence of the IR region. These assays confirmed that VarR was able to bind to the IR region, and the binding site was mapped using a series of DNA fragments that spanned the IR region (S3 Fig). VarR binds to the 30 bp *varR-varG* IR region that overlaps the -35 RNA polymerase transcriptional initiation sites for both *varG* and *varR* (Fig 5). Consistent with this interaction being highly specific, VarR was able to dissociate from the labelled 30 bp *varR-varG* IR DNA complex and bind to an unlabelled 30bp *varRG* IR DNA fragment during competitive EMSAs (Fig 5C), but did not bind a non-specific DNA sequence (Fig 5D). Therefore, VarR, by binding to this site, may negatively regulate its own transcription and that of the *varG*.

#### VarR acts as a repressor at the *varR-varG* promoter

Having identified that VarR binds at the *varR-varG* IR with high specificity, we then sought to determine its regulatory role at this site. As an LTTR, it was anticipated that VarR would repress transcription from the native *varRG* promoter, and thus that of the *varG* gene. In order to avoid the potential complication that β-lactams would be substrates for both VarG and VarR, the *varG* gene was substituted with the chloramphenicol resistance gene (*Cm*^*R*^) in constructs for antimicrobial susceptibility testing. Antimicrobial susceptibility testing, using the microdilution broth method, was performed using the following constructs pSMART/*varRG-Cm*^*R*^ and pSMART/*varR-varRG-Cm*^*R*^ in *E*. *coli* KAM3. The assays yielded substantial differences in the MICs between the two constructs tested (Table 5). For cells harboring the pSMART-*varRG-Cm*^*R*^ construct, there was a 64-fold increase in resistance (MIC, 128 μg/ml) for chloramphenicol compared to cells harboring pSMART/*varR-varRG-Cm*^*R*^ (MIC, 2 μg/ml), implying that VarR represses the transcription of the *Cm*^R^ gene at the *varRG* promoter. Consistent with this prediction, the addition (25 μg/ml) of penicillin to the cells haboring pSMART-*varR-varRG-Cm*^*R*^ caused an increase in their resistance to chloramphenicol (MIC, 32 μg/ml), implying that the β-lactam can de-repress expression of the *Cm*^*R*^ gene. To determine if this was a direct effect of the penicillin on VarR, we tested whether it could dissociate the VarR/*varRG*-DNA complex in EMSA assays; it was unable to do so (see https://figshare.com/s/d745d45927b941f8e4f2), implying that the de-repression is indirect.

**Table 5:** VarR regulation of *Cm*^*R*^ expression.

*E*. *coli* KAM3	Chloramphenicol MIC (μg/ml)
**pSMART only**	1
**pSMART/*varRG-Cm***^***R***^	128
**pSMART/*varR-varRG-Cm***^***R***^	2
**pSMART/*varR-varRG-Cm***^***R***^ ***+ 25 μg/ml penicillin***	32

#### VarR binds to the *varG-varA* intergenic region

**Fig 6:**
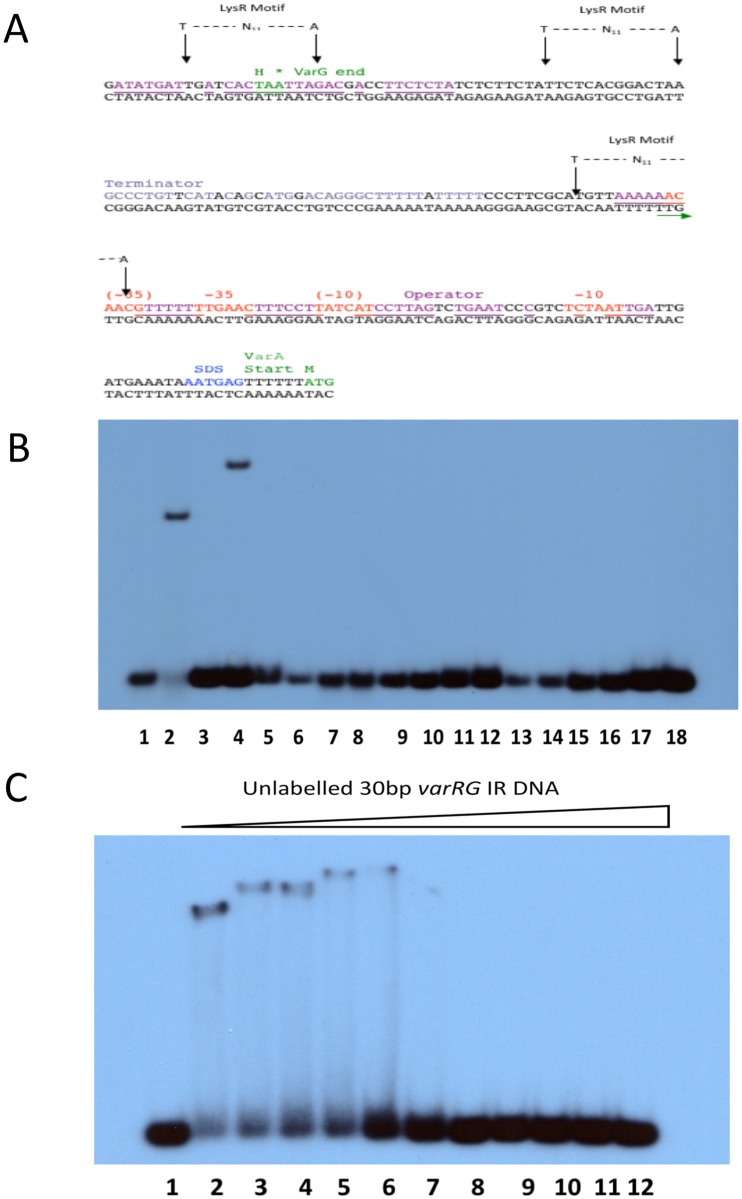
VarR binds to the *varG-varA* intergenic region. **(A)** The nucleotide sequence of the 176bp *varG-varA* intergenic region containing the putative promoter sequences represented by -35 and -10 regions (highlighted red). The Shine-Dalgarno sequence is highlighted blue. An operator site is highlighted in purple or underlined if overlapping with promoter sites. The terminator site is highlighted in grey. The start and stop codons and the deduced amino acid for VarA and VarG, respectively, are highlighted in green. **(B)** EMSA of VarR with *varG-varA* IR DNA. Lanes 1 and 2, 0 and 50 ng of VarR with 0.08 ng 30 bp *varR-varG* IR DNA (positive control). Lanes 3 to 18, VarR (0 and 50 ng, respectively) with 0.08 ng 30 bp *varG-varA* IR DNA fragments 1 to 8, respectively. VarR binds specifically to a 30 bp *varG-varA*1 IR DNA fragment, which incorporates the last 12 bp of the *varG* gene and the first 18 bp of the *varG-varA* IR. **(C)** Competitive EMSA of VarR/ 0.08 ng varG-varA1 DNA complex with unlabelled varR-varG IR DNA. Lane 1 and 2, 0 and 50 ng VarR with 0.08 ng varG-varA1 IR DNA, respectively. Lanes 3 to 12, competitive assay of 50 ng VarR/ 0.08 ng varG-varA1 IR DNA complex with titrations of unlabelled 30 bp varR-varG IR DNA (0.125, 0.25, 0.5, 1, 2, 4, 8, 16, 32, 64 ng, respectively).

A putative initiation codon for *varA* was identified 176 bp downstream of the stop codon for *varG* (Fig 6A). A palindromic self-complementary GC-rich region followed by a series of adenine nucleotides is located at the terminating sequences of *varG* and may form a hairpin that corresponds to a *rho*-independent terminator signal. The 176 bp *varG-varA* IR accommodates a putative promoter for *varA* with putative -10 and -35 sites and three T-N_11_-A motifs. Upstream from the initiation codon of *varA*, a likely promoter was found with a presumed -35 region (i.e. TTGATA) and a -10 region (i.e. TATCTT) separated by 15 bp. Like the *varR-varG* promoter, these regions consist of base pair substitutions that deviate from the general consensus sequence, which may affect promoter strength. Similar to the *varR-varG* IR, putative operator sequences have been identified which overlap the putative RNA polymerase initiation sites. Multiple putative -65 LTTR recognition-binding sites incorporating the T-N_11_-A motif were also identified for *varA*. The relative length of the IR could mean that VarR may form multimeric species to span the length of the promoter.

An EMSA analysis revealed that VarR binds to the *varGA* IR (Panel A in S4 Fig). To define the region to which VarR binds specifically in the 176 bp *varG-varA* IR, 30 bp DNA fragments that span the entire *varR-varG* IR were designed and used for EMSAs (Fig 6B). Interestingly, and unexpectedly, VarR binds specifically to a 30 bp *varG-varA*1 IR DNA fragment, which incorporates the last 12 bp of the *varG* gene and the first 18 bp of the *varG-varA* IR (Lane 4, Fig 6B). Using lower titrations of VarR indicated that the binding of VarR to the 25 bp *varG-varA1* IR DNA is specific (Panel B in S4 Fig). Furthermore, consistent with this interaction being highly specific, VarR was able to dissociate from the labelled 30 bp *varR-varA1* IR DNA complex and bind to an unlabelled 30 bp *varR-varA1* IR DNA fragment during competitive EMSAs (Panel C in S4 Fig). The binding of VarR at this site may negatively regulate transcription of the *varG* gene. The binding of VarR to the *varG-varA1* IR site was unexpected: the predicted binding site was expected to map to an area covered by the 30 bp *varG-varA*8 IR DNA fragment, which incorporates the putative -35 and -10 sites of the 176 bp *varG-varA* IR. Although, the reason why VarR binds to this region of *varG-varA* IR has yet to be established, it could be that VarR binds to multiple regions of this large promoter.

To define the specificity to which VarR binds to the 30 bp *varG-varA*1 IR compared to the *varR-varG* IR, a competitive assay was performed to determine the minimum concentration of unlabelled 30 bp *varR-varG* IR DNA that is required to dissociate (50 ng) VarR from (0.08 ng) labelled 30 bp *varGA*1 IR DNA. Fig 6C shows that VarR has an equal affinity for both the 30 bp *varR-varG* IR DNA and the 30 bp *varG-varA*1 IR DNA, with 1 ng of 30 bp *varR-varG* IR DNA sufficient to dissociate VarR from the complex. This is comparable with the competitive assays of VarR/ *varG-varA*1 IR DNA complex with unlabelled *varG-varA*1 IR DNA.

#### VarR binds to the *varB-varC* intergenic region

**Fig 7:**
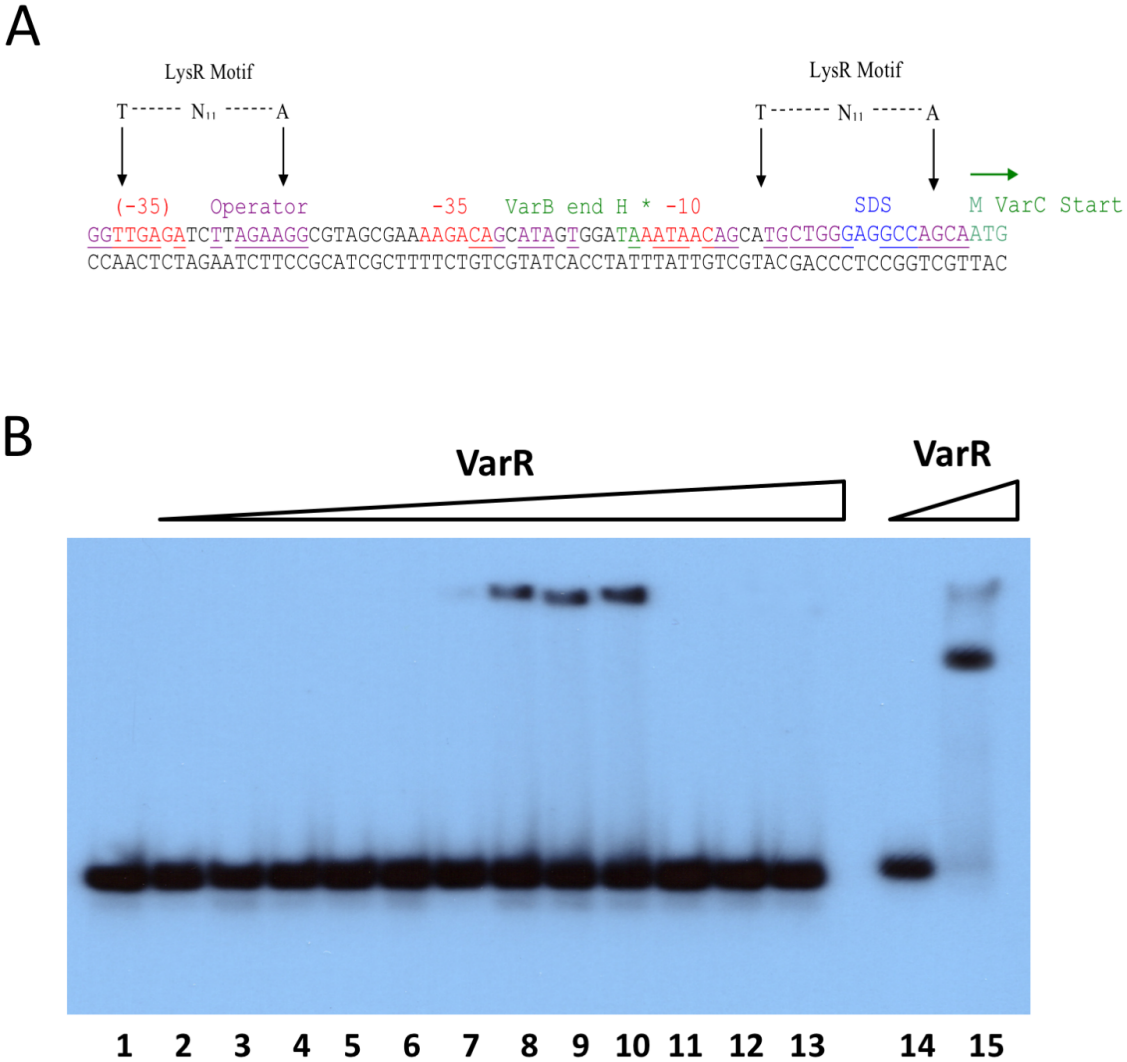
VarR binds to the varB-varC intergenic region. **(A)** The nucleotide sequence of the *varB-varC* intergenic region containing the putative promoter sequences represented by -35 and -10 regions (highlighted red). The Shine-Dalgarno sequence is highlighted blue. The start and stop codons and the deduced amino acid for VarC and VarB, respectively, are highlighted in green. An operator site is highlighted in purple or underlined if overlapping with promoter sites. **(B)** EMSA using increasing titrations of VarR with the 25 bp *varB-varC* IR DNA. Lanes 1 to 10, titrations of VarR (0, 1.25, 2.5, 5, 10, 25, 50, 100, 200, 400 ng, respectively) with 0.08 ng 25 bp *varB-varC* IR DNA. Lanes 11 to 13, titrations of VarR (0, 50 and 200ng, respectively) with 0.08ng 30 bp non-specific DNA (negative control). Lanes 14 and 15, 0 and 50 ng VarR with 0.08 ng 30 bp varR-varG IR DNA (positive control).

A putative initiation codon for *varC* was identified 25 bp downstream of the stop codon for *varB*. (Fig 7A). There is a putative promoter region upstream of the initiation codon, which appears to penetrate the terminal sequences of the *varB* gene, with -10 (i.e. AATAAC) and -35 (i.e. AAGACA) elements that are separated by 12 bp. A putative -65 LTTR recognition-binding site, incorporating a T-N_11_-A motif, lies within the 3’-end of *varB*. An EMSA analysis confirmed that VarR binds to the *varB-varC* IR (Fig 7B) and that binding of VarR to the 25 bp *varB-varC* IR is specific (Fig 7C). Furthermore, VarR was able to dissociate from the labelled 25 bp *varB-varC* IR DNA complex and bind to an unlabelled 25 bp *varB-varC* IR DNA fragment during competitive EMSAs (see https://doi.org/10.6084/m9.figshare.5346274.v1).

Considering that most efflux pumps involved in conferring antibiotic resistance are under the control of transcriptional repressors that bind the same substrates as the pump, leading to de-repression of the pump genes, we sought to test and found that the macrolide erythromycin could not dissociate VarR from the *varG-varA* and *varB-varC* intergenic regions (see https://doi.org/10.6084/m9.figshare.5346400.v1).

## Discussion

We have identified a novel resistance regulon, in which a metallo-β-lactamase, with high activity against carbapenems, and an efflux pump, capable of extruding macrolides, have been brought together under the control of a LysR family transcriptional activator in the pathogenic bacteria *Vibrio cholerae*. The association of efflux pumps with other resistance determinants has been observed previously; recent reports suggest that, they not only provide co-resistance, but can enhance the resistance provided by other determinants, increasing resistance levels, which can have a serious impact on antimicrobial therapy in the clinical setting [69]. Efflux pumps not only expel a broad range of antibiotics owing to their poly-substrate specificity, but also drive the acquisition of additional resistance mechanisms by lowering intracellular antibiotic concentrations and promoting mutation accumulation. For example, recent studies indicate that the expression of efflux pumps increases the resistance to β-lactams, afforded by β-lactamases, in both *Pseudomonas aeruginosa* [18,70] and *Klebsiella pneumonia* [71]. Although these studies revealed an interaction in these resistance determinants; to our knowledge, our study is the first report of a system where both a β-lactamase and an efflux pump occur in the same regulon and are regulated by the same transcriptional regulator.

Carbapenem antibiotics are often considered as the last resort drugs for treating a wide range of bacterial infections [72]. However, resistance to carbapenems can occur and is often multifactorial, arising from the synergistic effects of a decrease in the expression of outer-membrane porins (OMP) that facilitate carbapenem entry into cells; the overexpression of tripartite pumps, responsible for carbapenem extrusion; and, most importantly, the production of metallo-β-lactamases, which can degrade carbapenems [18,73,74]. Indeed, Enterobacteriacaea, such as *Klebsiella pneumonia*, that can produce metallo-β-lactamases, such as NDM-1 [75], have emerged worldwide as important pathogens of nosocomial infections because of the difficulty in treating the infections they cause [16,72]. Carbapenems are also an important therapeutic for treating infections due to *Pseudomonas aeruginosa*, because they are effective when other β-lactams are not due to production of the serine β-lactamase AmpC [76]. However, resistance can still occur due to down-regulation of the OMP OprD and/or up-regulation of the MexAB-OprM tripartite efflux pump [18,19,73]. It has been reported that β-lactamase, AmpC, resistance is coupled to other MDR mechanisms such as efflux via MexAB-OprM in *P*. *aeruginosa* [18,19,77]. However, these have not been explicitly linked as being co-expressed from the same operon or by the same regulatory system. Rather resistance is initiated by the presence of substrate or mutation in a regulatory gene, MexR, resulting in over-expression of the MexAB-OrpM efflux pump [77,78]. The Var resistance determinant is novel in that a β-lactamase and an efflux pump occur in the same regulon and are apparently co-regulated by the same transcription factor, VarR. It is interesting though to note that the *varG*, *varAB* and *varCDEF* genes are not organized into a single operon; but instead organized as a regulon, with binding sites for VarR in each of the IRs preceding these genes. This would suggest that expression of these genes is differentially regulated.

The Var resistance determinant may have developed for, and has the potential to provide, high-level resistance to carbapenems, by enabling both their degradation and extrusion from the cell. The use of an LTTR to regulate the regulon would be consistent with this system having evolved a primary role in β-lactam, and specifically carbapenem, resistance. Indeed, penicillin G was able to de-repress expression of VarG, but not by directly inducing the release of VarR from the *varG* promoter. The arrangement of the *varR* and *varG* genes is strikingly similar to the well-documented *ampR-ampC* system, composed of the LTTR AmpR and the serine β-lactamase AmpC, which is found in many pathogenic bacteria [8–11]. The mechanism of AmpC induction by AmpR is a complex process that is intricately linked to peptidoglycan (or murein) cell wall recycling [8–10,79–81]. Bacterial peptidoglycan is a dynamic surface and is continuously remodelled through synthesis and degradation as the bacterium grows and divides [82,83]. Bactericidal β-lactam antibiotics were developed specifically to disrupt this balance, which has ultimately led to the evolution of bacterial defence mechanisms, such as β-lactamase production against these agents [82]. AmpC synthesis is therefore activated in the presence of β-lactam antibiotics as a result of AmpR derepression. However, β-lactams, which are not known to enter the cytoplasm [84], do not directly activate AmpR [79]. This mechanism involves disruption of the peptidoglycan by β-lactams that leads to increased periplasmic accumulation of cell wall precursors and degradation products (muropeptides). A cytoplasmic membrane permease, AmpG, has specificity for these muropeptides and transports them into the cytoplasm. Within the cytoplasm, muropeptides derepress AmpR leading to induction of AmpC [82,85]. A cytosolic protein, AmpD, encoded on a different operon from the *ampR and ampC* genes [86], is responsible for degradation of these muropeptides, regenerating precursors for peptidoglycan synthesis. However, both AmpD and AmpG are also essential for the regulation of β-lactamase activity [87]. A mutation in *ampD* leads to an accumulation of muropeptides that continually activates AmpR leading to continued AmpC expression [86–90]. Mutational inactivation of AmpG renders the bacterium AmpC non-inducible due to the absence of these activating muropeptides [81,87,91]. This demonstrates a close association between β-lactamase induction and peptidoglycan recycling [87]. Similarly, muropeptides could bind to VarR to induce expression of the VarG β-lactamase. Our experiments revealed that the expression of *Cm*^*R*^ is blocked from a *varR-IR*_*varRG*_*-Cm*^*R*^ construct, presumably due to VarR binding to the *varR-varG* IR, but is expressed when cells with this construct are exposed to penicillin G. Since penicillin G would not be expected to penetrate the cytoplasm, and our studies confirmed that it is incapable of directly interacting with VarR to cause its release from the *varR-varG* IR, the derepression caused by penicillin G must be indirect, possibly by inhibiting peptidoglycan synthesis. Why has such a resistance determinant become linked to an efflux pump? The MexAB-OprM pump in *P*. *aeruginosa* has been shown to confer resistance to meropenem and other β-lactams [92] and that this arises due to meropenem interacting with the RND transporter MexB [93]. It is conceivable that the VarG Mβl and the VarACDEF pump have become linked to give enhanced resistance to β-lactams, with these also being extruded by the pump. It is notable that the VarD and VarE proteins are predicted to possess large periplasmic domains, similar to those found in RND transporter, which could provide the binding sites for β-lactams and catalyzing their removal from the periplasm. However, we were unable to establish that the efflux pump confers resistance to meropenem. Further work will be necessary to define if VarR is similarly regulated by muropeptides; and specifically, if other substrates act as co-effectors differentially regulating the interaction of VarR with the *varRG*, *varGA* and *varBC* IRs. Recent studies revealed a group of MF drug transporters that are also apparently regulated by an upstream LysR type transcription factors [23], leading to the suggestion that such pumps are regulated by additional factors, possibly secreted by the host.

The coupling of enzymatic and efflux resistance mechanism in the *var* regulon is of concern because it could also provide co-resistance that would undermine the use of other antibiotics in combination with β-lactams. In the case of carbapenem-resistant *K*. *pneumoniae*, these strains are still susceptible to a few antibiotics, such as tigercycline and the cyclic peptide polymixin, but they can utilize tripartite pumps, such as the AcrABTolC pump, to confer resistance to both of these drugs [94]. A sequence analysis revealed that both VarD and VarE resemble ABC-type permeases that confer resistance to macrolides and antimicrobial peptides. Our studies revealed that the VarACDEF pump can confer resistance to a range of substrates, including macrolides, which, like tigercycline, target the bacterial ribosome. Consequently, the possibility of the *var* resistance determinant arising in other bacterial species, by horizontal gene transfer, is also of concern. *V*. *cholerae* and enteric bacteria can both reside within the intestinal tract, providing an opportunity for horizontal gene transfer between strains.

## Supporting information

S1 Table The oligonucleotides used in this study. (DOCX)

S2 Table Plasmids used in this study. (DOCX)

S3 Table Bacterial strains used in this study. (DOCX)

S4 Table The IC50 of *ΔtolC* mutant *E*.*coli* TG1, harbouring the plasmid encoding *varDEF*, to macrolides and quinolones. (DOCX)

S1 Fig The metallo- β-lactamase VarG forms dimers. **The SEC elution profile for VarG.** (A) The elution profile revealed two peaks, the first peak (with an elution volume of 70.07 ml) corresponding to the putative VarG dimer and the second peak (with an elutyion volume of 78.86 ml) to the monomer. Samples from the two peaks ran at identical positions on an SDS-PAGE gel and were confirmed by mass spectrometry as VarG. **Analytical ultracentrifugation analyses of the oligomeric state of VarG**. SV AUC experiments were performed at a rotor speed of 42,000 rpm at 20°C. Plots of *c(s*, *f*_*r*_*) and c(s*, *M)* were generated by MATLAB 7.0 software. The calculated *c(s*, *f*_*r*_*)* distribution is plotted in two dimensions with grid lines representing the *s* and *f*_*r*_ grids in the thermograph (B). A contour plot from the *c(s*, *f*_*r*_*)* surface was projected into the *s-f*_*r*_ plane, where the magnitude of *c(s*, *f*_*r*_*)* is indicated by the contour lines at constant *c(s*, *f*_*r*_*)*, in equidistant intervals of *c*. *c(s*, *M)*, and the distribution was transformed from the calculated *c(s*, *f*_*r*_*)* distribution (C). The dotted lines indicated lines of *f*_*r*_ (frictional ratio). The signal of the *c*(*s*,*M*) distribution is indicated by the color temperature (C). This SV AUC analysis indicated that VarG forms dimers, with a calculated molecular mass of 86.9 kDa. SE AUC experiments were performed at rotor speeds of 16,000, 20,000, 24,000, 28,000, and 32,000 rpm at 20°C. The experimental data was analyzed by SEDPHAT software and the data is shown as a plot of sample signal versus its distance from the center of rotation at each rotor speed (D). This SE AUC analysis indicated that VarG forms dimers with a predicted molecular mass of 75.8 kDa. **Deconvolution high resolution ESI mass spectrum of the putative VarG dimer from SEC**. *n* proton masses have been subtracted from each (M+nH)^n+^ ion to yield the corresponding zero-charge mass in the deconvolved spectrum. This analysis (using 50 μg/mL of protein) revealed a major peak, with a molecular mass of 41,679 Da, and a minor peak, with a molecular mass of 83,359 Da protein, corresponding to the VarG monomer and dimer.
(TIFF)

S2 Fig VarF is an ATPase. The ATPase activity of VarF was determined using a malachite green assay to monitor Pi production. The rate of ATP hydrolysis was measured as a function of the ATP concentration and the data fitted to a sigmoidal equation, indicating values for the V_max_, K_m_ and Hill Coefficient of 34.6 (± 2.3) nmoles Pi/min/mg, 795 (± 81.2) μM and 1.87 (± 0.29), respectively.
(TIFF)

S3 Fig EMSA analysis of VarR binding to *varR-varG* IR. EMSA analysis of VarR binding to *varR-varG* IR with (0.08 ng) 302 bp, 1^st^ and 2^nd^ 151 bp, 1^st^ 31 bp, 2^nd^ 32 bp of the *varR-varG* IR. Titrations of VarR (0, 50, 200ng, respectively) with 0.08ng of 302 bp *varR-varG* IR (Lanes 1 to 3), 1^st^ 151 bp *varR-varG* IR (Lanes 4 to 6), 2^nd^ 151 bp *varR-varG* IR (Lanes 7 to 9), 1^st^ 31 bp *varR-varG* IR (Lanes 10 to 12), and 2^nd^ 31 bp *varR-varG* IR (13 to 15). Lanes 16 and 17, 0.08ng 31bp Mtr1 DNA with 0ng and 200ng MtrR, respectively (positive control). Retardation of the 302 bp, the 1^st^ and 2^nd^ 151 bp and the 2^nd^ 32 bp *varR-varG* IR DNA fragments following incubation with 50 and 200ng VarR, respectively. However, the 1^st^ 31bp *varR-varG* IR was not retarded by VarR.
(TIFF)

S4 Fig EMSA analysis of VarR binding to *varG-varA* IR. **(A)** EMSA analysis of VarR binding to *varG-varA* IR with 415 bp, 1^st^ 207 bp, 2^nd^ 208 bp, 176 bp, 1^st^ and 2^nd^ 88 bp of the *varG-varA* IR. Lanes 1 and 2, VarR (0 ng and 50 ng, respectively) with 0.08 ng 30 bp *varR-varG* IR DNA (positive control). VarR (0, 50, 100 ng, respectively) with 415 bp (Lanes 3 to 5), 1^st^ 207 bp (Lanes 6 to 8), 2^nd^ 208 bp (Lanes 9 to 11), 176 bp (Lanes 12 to 14), 1^st^ 88 bp (lanes 15 to 17) and 2^nd^ 88 bp *varGA* IR DNA (lanes 18 to 20). Retardations with 0.08 ng 415 bp, 1^st^ 207 bp, 2^nd^ 208 bp, 176 bp, 1^st^ and 2^nd^ 88 bp *varG-varA* IR DNA fragments following incubation with 0, 50, 200ng VarR, respectively, are observed. **(B)** EMSA using increasing titrations of VarR with 30 bp *varG-varA*1 IR DNA including 30 bp non-specific DNA. Lanes 1 and 2, VarR (0 and 50 ng) with 0.08 ng 30 bp *varRG* IR DNA (positive control). Lanes 3 to 11, titrations of VarR (0, 1.25, 2.5, 5, 10, 25, 50, 100, 200 ng, respectively) with 0.08 ng 30 bp *varG-varA*1 IR DNA. Lanes 12 to 17, titrations of VarR (0, 5, 10, 50, 100, 200 ng, respectively) with 0.08 ng 30 bp non-specific DNA. **(C)** Competitive EMSA of VarR/ 0.08 ng *varG-varA1* DNA complex with unlabelled *varR-varG* IR DNA. Lane 1 and 2, 0 and 50 ng VarR with 0.08 ng *varG-varA1* IR DNA, respectively. Lanes 3 to 12, competitive assay of 50 ng VarR/ 0.08 ng *varG-varA1* IR DNA complex with titrations of unlabelled 30 bp *varR-varG* IR DNA (0.125, 0.25, 0.5, 1, 2, 4, 8, 16, 32, 64 ng, respectively).
(TIFF)

S5 Fig VarR binds to the *varB-varC* IR. EMSA of VarR with 195 bp, 1^st^ 97 bp, 2^nd^ 98 bp and 25 bp of the *varB-varC* IR. Titrations of VarR (0, 50 and 200 ng, respectively) with 0.08 ng 195 bp *varB-varC* IR DNA (Lanes 1 to 3), 1^st^ 97 bp *varB-varC* IR DNA (Lanes 4 to 6), 2^nd^ 98 bp *varB-varC* IR DNA (Lanes 7 to 9), and 25 bp *varB-varC* IR DNA (Lanes 10 to 12). Lanes 13 and 14, 0 ng and 50 ng VarR with 0.08 ng 30 bp *varR-varG* IR DNA (positive control), respectively. Retardations with 0.08 ng 195 bp, 1^st^ 97 bp, 2^nd^ 98 bp and 25 bp *varB-varC* IR DNA fragments following incubation with 50 and 200 ng VarR are observed.
(TIFF)
