# Supplementary material for: The Vibrio cholerae var regulon encodes a metallo-β-lactamase and an antibiotic efflux pump, which are regulated by VarR, a LysR-type transcription factor
Source: PLoS One. 2017 Sep 12;12(9):e0184255. doi: 10.1371/journal.pone.0184255 (PMC5595328; doi:10.1371/journal.pone.0184255)
Supplement: S1 Table — (DOCX) [file pone.0184255.s001.docx]

**S1 Table - Oligonucleotides used in this study.**

| **Oligonucleotide** | | | **Sequence** | | | |  |
| --- | --- | --- | --- | --- | --- | --- | --- |
| **Var1** | | | | 5’-P-CTCATCACCTAATAATTTCGTGCGTAAATG-3’ | | | |
| **Var2** | | | | 5’-P-TTAGTGATCAATCATATCTGCGAGAATCATTGC-3’ | | | |
| **Var3** | | | | 5’-GGATCCGTCGCCCCCGAAATCAATTTGCGCAGC-3’ | | | |
| **Var4** | | | | 5’-AAGCTTTTATTTCTTATCGATCTCTTCATAGAC-3’ | | | |
| **Var5** | | | | 5’-AACCCCGAATTCGAAAGGCCATGACCACGACAATC-3’ | | | |
| **Var6** | | | | 5’-AACCCCCTGGAGGTGATCAATCATATCTGCGAGAATC-3’ | | | |
| **Var7** | | | | 5’-CCATGGGCAAGTCTTTATCTCCTGCTCCTTTGGTG-3’ | | | |
| **Var8** | 5’-AGATCTTCAATGATGATGATGATGATGAGCAGTGGGCT CATCACTGACGACTTG-3’ | | | | |  |  |
| **Var9** | 5’-AGCAGAGAGCTCCGCCTCTTGTTACAGCAATCTTG-3’ | | | | |  |  |
| **Var10** | 5’-ATAAACCCCGGGTTAAGCAGTGGGCTCATCACTGA-3’ | | | | |  |  |
| **Var11** | 5’-GGGGCCCATATGTTGATTGATGAAATAAATGAGTTTTTTATGGC-3’ | | | | |  |  |
| **Var12** | 5’-AAAAAAGCGGCCGCAGCAGTGGGCTCATCACTGA-3’ | | | | |  |  |
| **Var13** | 5’- GGTTGGCTCATATTCAATGCTTGTGCGGCT -3’ | | | | |  |  |
| **Var14** | 5’–GCCTTTAGCACTTGCATTAAAAGTAAGTAG-3’ | | | | |  |  |
| **Var15** | 5’–TCATTTACGCACGAAATTATTAGGTGATGA–3’ | | | | |  |  |
| **Var16** | 5’–TATACATACTATAACAAGCATGCATTTTTG-3’ | | | | |  |  |
| **Var17** | 5’–CTCATCACCTAATAATTTCGTGCGTAAATGA-3’ | | | | |  |  |
| **Var18** | 5’–TCATTTACGCACGAAATTATTAGGTGATGAG-3’ | | | | |  |  |
| **Var19** | 5’–TATACATACTATAACAAGCATGCATTTTTGAT-3’ | | | | |  |  |
| **Var20** | 5’–ATCAAAAATGCATGCTTGTTATAGTATGTATA-3’ | | | | |  |  |
| **Var21** | 5’–AGATCACTAACAATGTACATACCGCACTGG–3’ | | | | |  |  |
| **Var22** | 5’–CCGGCTGACGTTGAGGGTCGGTAATGGGGA-3’ | | | | |  |  |
| **Var23** | 5’–ATGCGAAGGGAAAAATAAAAAGCCCTGTCC–3’ | | | | |  |  |
| **Var24** | 5’–GTTAAAAAACAACGTTTTTTTGAACTTTCC–3’ | | | | |  |  |
| **Var25** | 5’–TTAGACGACCTTCTCTATCTCTTCTATTCT–3’ | | | | |  |  |
| **Var26** | 5’–AAAAAACTCATTTATTTCATCAATCAATTA-3’ | | | | |  |  |
| **Var27** | 5’–ATGCGAAGGGAAAAATAAAAAGCCCTGTCC-3’ | | | | |  |  |
| **Var28** | 5’–GTTAAAAAACAACGTTTTTTTGAACTTTCC-3’ | | | | |  |  |
| **Var29** | 5’-GATATGATTGATCACTAATTAGACGACCTT-3’ | | | | |  |  |
| **Var30** | 5’-AAGGTCGTCTAATTAGTGATGAATCATATC-3’ | | | | |  |  |
| **Var31** | 5’–GCGTACAGGTTGAGATCTTAGAAGGCGTAG-3’ | | | | |  |  |
| **Var32** | 5’–TGTGTGCATCGGGTGATTGTTGCTTGGCTT-3’ | | | | |  |  |
| **Var33** | 5’–TGCGGTGAGAAAGCCCTTATTCATGCTGGC-3’ | | | | |  |  |
| **Var34** | 5’–TGTGTACTGACTAGCCTGATGGCATCTTCT-3’ | | | | |  |  |
| **Var35** | 5’–ATAACAGCATGCTGGGAGGCCAGCA-3’ | | | | |  |  |
| **Var36** | 5’–TGCTGGCCTCCCAGCATGCTGTTAT–3’ | | | | |  |  |
| **Var37** | 5’-TCTAGATTATTTCTTATCGATCTCTTCATAGACTAA-3’ | | | | |  |  |
| **Var38** | 5’-CATATGATCAAAAATGCATGCTTGTTATAGTAT-3’ | | | | |  |  |
| **Var39** | 5’-CATATGGAGAAAAAAATCACTGGATATACCACC-3’ | | | | |  |  |
| **Var40** | 5’-CTCGAGTTACGCCCCGCCCTGCCACTCATCGCA-3’ | | | | |  |  |
| **Var41** | 5’-P-CTCATCACCTAATAATTTCGTGCGTAAATG-3’ | | | | |  |  |
| **Var42** | 5’-P-TTACGCCCCGCCCTGCCACTCATCGCAGTA-3’ | | | | |  |  |
| **Var43** | 5’-P-TTATTTCTTATCGATCTCTTCATAGACTAAATTTTT-3’ | | | | |  |  |
| **30bp *varGA*1 IR**  varGA1Fwd  varGA1Rev | | 5’-GATATGATTGATCACTAATTAGACGACCTT-3’  5’-AAGGTCGTCTAATTAGTGATGAATCATATC-3’ | | |  |  |  |
| **30bp *varGA*2 IR**  varGA2Fwd  varGA2Rev | | 5’-CTCTATCTCTTCTATTCTCACGGACTAAGC-3’  5’-GCTTAGTCCGTGAGAATAGAAGAGATAGAG-3’ | | |  |  |  |
| **30bp *varGA*3 IR**  varGA3Fwd  varGA3Rev | | 5’-CCTGTTCATACAGCATGGACAGGGCTTTTT-3’  5’-AAAAAGCCCTGTCCATGCTGTATGAACAGG-3’ | | |  |  |  |
| **30bp *varGA*4 IR**  varGA4Fwd  varGA4Rev | | 5’-ATTTTTCCCTTCGCATGTTAAAAAACAACG-3’  5’-CGTTGTTTTTTAACATGCGAAGGGAAAAAT-3’ | | |  |  |  |
| **30bp *varGA*5 IR**  varGA5Fwd  varGA5Rev | | 5’-TTTTTTTGAACTTTCCTTATCATCCTTAGT-3’  5’-ACTAAGGATGATAAGGAAAGTTCAAAAAAA-3’ | | |  |  |  |
| **30bp *varGA*6 IR**  varGA6Fwd  varGA6Rev | | 5’-CTGAATCCCGTCTCTAATTGATTGATGAAA-3’  5’-TTTCATCAATCAATTAGAGACGGGATTCAG-3’ | | |  |  |  |
| **30bp *varGA*7 IR**  varGA7Fwd  varGA7Rev | | 5’-TAAATGAGTTTTTTATGGCAAAACGTTGGA-3’  5’-TCCAACGTTTTGCCATAAAAAACTCATTTA-3’ | | |  |  |  |
| **30bp *varGA*8 IR**  varGA8Fwd  varGA8Rev | | 5’-AACAACGTTTTTTTGAACTTTCCTTATCAT-3’  5’-ATGATAAGGAAAGTTCAAAAAAACGTTGTT-3’ | | |  |  |  |
| **31bp Positive-control**  VarPC-Fwd  VarPC-Rev | | 5’-TTTTTATCCGTGCAATCGTGTATGTATAATG -3’  5’-CATTATACATACACGATTGCACGGATAAAAA -3’ | | |  |  |  |
| **30bp Negative-control**  VarNC-Fwd  VarNC-Rev | | 5-ATTCCCGTTTCAGTTGACTTGCGACCAGCG-3’  5’-CGCTGGTCGCAAGTCAACTGAAACGGGAAT-3’ | | |  |  |  |
