# Supplementary material for: The Vibrio cholerae var regulon encodes a metallo-β-lactamase and an antibiotic efflux pump, which are regulated by VarR, a LysR-type transcription factor
Source: PLoS One. 2017 Sep 12;12(9):e0184255. doi: 10.1371/journal.pone.0184255 (PMC5595328; doi:10.1371/journal.pone.0184255)
Supplement: S2 Table — (DOCX) [file pone.0184255.s002.docx]

**S2 Table – Plasmids used in this study**

| **Plasmid** | **Description** | **Source/ Reference** |
| --- | --- | --- |
| pGEM-T Easy | Cloning vector compatible with α-complementation, Ap^R^ | Promega |
| pSMART HC-Kan | High copy cloning vector for expression of proteins from native promoters, Kn^R^ | Lucigen |
| pET21b(+) | T7*lac* expression vector for C-terminal His_6_ tagged proteins, Ap^R^ | Novagen |
| pET28b(+) | T7*lac* expression vector for N- or C-terminal His_6_ tagged proteins, Kn^R^ | Novagen |
| pET33b(+) | T7*lac* expression vector for C-terminal His_6_ tagged proteins, Kn^R^ | Novagen |
| pQE-100 | T5 expression vector for N-terminal His_6_ tagged proteins, Ap^R^ | Qiagen |
| pREP4 | Expression of *lac* repressor protein encoded by *lacI* gene for highly regulated co-expression with pQE series of vectors, Kn^R^ | Qiagen |
| pBAD/ *Myc*-His B | Dose-dependent expression vector for C-terminal His_6_ tagged proteins, Ap^R^ | Invitrogen |
| pETDuet^TM^-1 | T7*lac* expression vector for the co-expression of multiple N-terminal His_6_ tagged and C-terminal S tagged proteins, Ap^R^ | Novagen |
| pACYCDuet^TM^-1 | T7*lac* expression vector for the co-expression of multiple N-terminal His_6_ tagged and C-terminal S tagged proteins, Cm^R^ | Novagen |
| pRSFDuet^TM^-1 | T7*lac* expression vector for the co-expression of multiple N-terminal His_6_ tagged and C-terminal S tagged proteins, Kn^R^ | Novagen |
| pCDFDuet^TM^-1 | T7*lac* expression vector for the co-expression of multiple N-terminal His_6_ tagged and C-terminal S tagged proteins, Sm^R^ | Novagen |
