# Supplementary material for: The Vibrio cholerae var regulon encodes a metallo-β-lactamase and an antibiotic efflux pump, which are regulated by VarR, a LysR-type transcription factor
Source: PLoS One. 2017 Sep 12;12(9):e0184255. doi: 10.1371/journal.pone.0184255 (PMC5595328; doi:10.1371/journal.pone.0184255)
Supplement: S3 Table — (DOCX) [file pone.0184255.s003.docx]

**S3 Table – Bacterial strains used in this study.**

| **Strain** | **Genotype** | **Application** | **Source/ Reference** |
| --- | --- | --- | --- |
| ***Escherichia coli*** | | | |
| NovaBlue | *end*A1 *hsd*R17 (r_k12-_m_k12+_) *sup*E44 *thi-*1 *rec*A1 *gyr*A96 *rel*A1 *lac* [F’*pro*A^+^B^+^ *lac*I^q^ZΔM15::Tn*10*(tet^R^)] | General cloning | Novagen |
| E. cloni | F-*mcr*A Δ (*mrr-hsd*RMS-*mcr*BC)  Φ80*dlac*ZΔM15 Δ*lac*X74  *end*A1 *rec*A1*ara*D139  Δ(*ara, leu*)7697 *gal*U *gal*K *rps*L *nup*G λ- *ton*A | General cloning | Lucigen |
| BL21 (DE3) | F^-^ *omp*T *hsd*S_B_(r_B_^-^m_B_) *gal dcm met* (DE3) | Protein expression | Stratagene |
| BL21 Star | F^-^ *omp*T *hsd*S_B_(r_B_^-^m_B_) *gal dcm rne*1 31 (DE3) | Protein expression | Invitrogen |
| BL21(AI) | F^-^ *omp*T *hsd*S_B_(r_B_^-^m_B_) *gal dcm ara*B::*T7RNA-tet*A | Protein expression | Invitrogen |
| M15 | F^-^ *lac ara gal mtl recA uvr* | Protein expression | Qiagen |
| TOP10 | F-*mcr*A Δ (*mrr-hsd*RMS-*mcr*BC)  Φ80*dlac*ZΔM15 Δ*lac*X74  *deo*R *rec*A1*ara*D139  Δ(*ara*A*- leu*)7697 *gal*U *gal*K *rps*L *end*A *nup*G | General cloning | Invitrogen |
| LMG194 | F^-^ Δ*lacX74 gal E thi rpsL Δpho*A (*pvu* II) Δ*ara*714 *leu*::Tn*10* | Protein expression | Invitrogen |
| XL10-Gold | Tet^R^ Δ (*mcr*A) 183 Δ (*mcr*CB-*hsd*SMR-*mrr*) 173 *end*A1 *sup*E44 *thi*-1 *rec*A1 *gyr*A96 *rel*A1 *lac* Hte [F’ *pro*AB *lacl*^q^ZΔM15 Tn10 (Tet^R^) Amy Cam^R^]^a^ | Site directed mutagenesis | Stratagene |
| C43 (DE3) | F^-^ *omp*T *hsd*S_B_(r_B_^-^m_B_) *gal dcm* (DE3) | Protein expression | [1] |
| KAM3 (DE3) | Δ*acrAB* | Complementation analysis | [2] |
| N43 (DE3) | F^-^*lac ara mal xyl mtl gal rpsL acrA*1 *tolC*::Tn*10* | Complementation analysis | [3] |
| TG1 (DE3) | Δ*tolC* | Complementation analysis | [4] |
| ***Vibrio cholerae*** | | | |
| CVD101 | CT-A^-^, CT-B+ deletion derivative of classical biotype Ogawa serotype 395 | Source of chromosomal DNA | [5] |

**References:**

[1] Miroux B, Walker JE. (1996) Over-production of proteins in *Escherichia co*li: mutant hosts that allow synthesis of some membrane proteins and globular proteins at high levels. J Mol Biol. 260:289-98.

[2] Morita Y, Kodama K, Shiota S, Mine T, Kataoka A, Mizushima T, Tsuchiya T. (1998) NorM, a putative multidrug efflux protein, of *Vibrio parahaemolyticus* and its homolog in *Escherichia coli*. Antimicrob Agents Chemother. 42:1778–1782.

[3] Ma D, Cook DN, Alberti M, Pon NG, Nikaido H, Hearst JE. (1993) Molecular cloning and characterization of *acrA* and *acr*E genes of *Escherichia coli*. J Bacteriol. 175:6299-313.

[4] Nagakubo S, Nishino K, Hirata T, Yamaguchi A. (2002) The putative response regulator BaeR stimulates multidrug resistance of *Escherichia coli* via a novel multidrug exporter system, MdtABC. J Bacteriol.  184:4161–4167.

[5] Levine MM, Kaper JB, Herrington D, Losonsky G, Morris JG, Clements ML, Black RE, Tall B, Hall R. (1988) Volunteer studies of deletion mutants of *Vibrio cholerae* O1 prepared by recombinant techniques. Infect Immun. 56:161-7.
