# Supplementary material for: The Vibrio cholerae var regulon encodes a metallo-β-lactamase and an antibiotic efflux pump, which are regulated by VarR, a LysR-type transcription factor
Source: PLoS One. 2017 Sep 12;12(9):e0184255. doi: 10.1371/journal.pone.0184255 (PMC5595328; doi:10.1371/journal.pone.0184255)
Supplement: S4 Table — (DOCX) [file pone.0184255.s004.docx]

**S4 Table – The IC_50_ of *△tolC* mutant *E. coli* TG1, harbouring the plasmid encoding *varDEF,* to macrolides and quinolones.**

|  | IC_50_ (μg/mL) | |  |
| --- | --- | --- | --- |
| Drug group and drug | TG1/pQE100  (Control) | TG1/pQE100-*varDEF* | Relative resistance^a^ |
| **Macrolide** |  |  |  |
| Azithromycin | 0.9 | 0.45 | 0.5 |
| Clarithromycin | 0.8 | 0.4 | 0.5 |
| Erythromycin | 0.8 | 0.8 | 1 |
| Spiramycin | 3.45 | 1.725 | 0.5 |
| **Quinolone** |  |  |  |
| Norfloxacin | 0.007 | 0.007 | 1 |
| Ciprofloxacin | 0.003 | 0.003 | 1 |
| Ofloxacin | 0.06 | 0.03 | 0.5 |

^a^Relative resistance is the ratio of the IC_50_ for TG1/pQE100-varDEF to the IC_50_ for TG1/pQE100
