# Supplementary figures and images for: The Vibrio cholerae var regulon encodes a metallo-β-lactamase and an antibiotic efflux pump, which are regulated by VarR, a LysR-type transcription factor
Source: PLoS One. 2017 Sep 12;12(9):e0184255. doi: 10.1371/journal.pone.0184255 (PMC5595328; doi:10.1371/journal.pone.0184255)

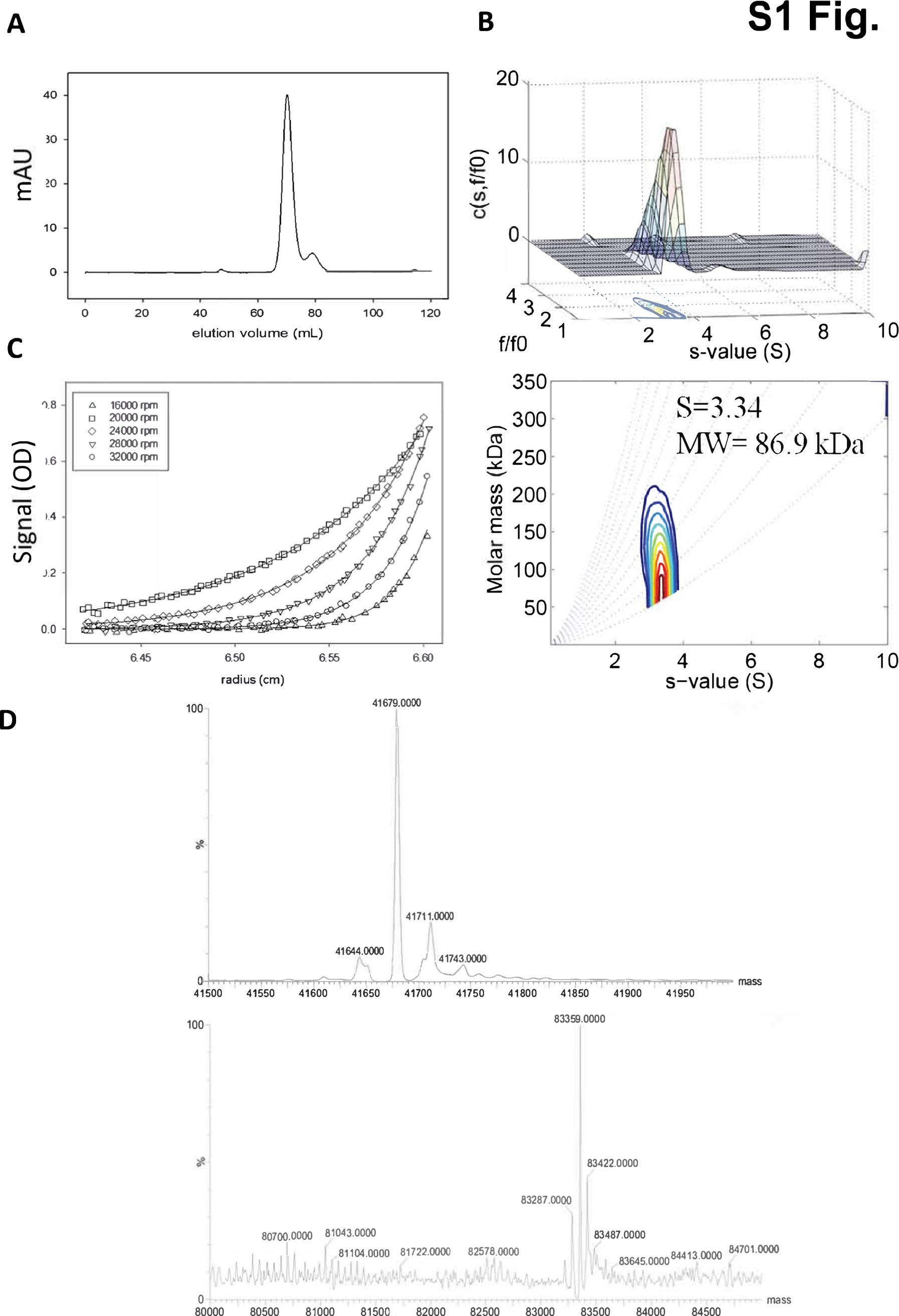

Supplement: S1 Fig — The SEC elution profile for VarG. (A) The elution profile revealed two peaks, the first peak (with an elution volume of 70.07 ml) corresponding to the putative VarG dimer and the second peak (with an elutyion volume of 78.86 ml) to the monomer. Samples from the two peaks ran at identical positions on an SDS-PAGE gel and were confirmed by mass spectrometry as VarG. Analytical ultracentrifugation analyses of the oligomeric state of VarG. SV AUC experiments were performed at a rotor speed of 42,000 rpm at 20°C. Plots of c(s, fr) and c(s, M) were generated by MATLAB 7.0 software. The calculated c(s, fr) distribution is plotted in two dimensions with grid lines representing the s and fr grids in the thermograph (B). A contour plot from the c(s, fr) surface was projected into the s-fr plane, where the magnitude of c(s, fr) is indicated by the contour lines at constant c(s, fr), in equidistant intervals of c. c(s, M), and the distribution was transformed from the calculated c(s, fr) distribution (C). The dotted lines indicated lines of fr (frictional ratio). The signal of the c(s,M) distribution is indicated by the color temperature (C). This SV AUC analysis indicated that VarG forms dimers, with a calculated molecular mass of 86.9 kDa. SE AUC experiments were performed at rotor speeds of 16,000, 20,000, 24,000, 28,000, and 32,000 rpm at 20°C. The experimental data was analyzed by SEDPHAT software and the data is shown as a plot of sample signal versus its distance from the center of rotation at each rotor speed (D). This SE AUC analysis indicated that VarG forms dimers with a predicted molecular mass of 75.8 kDa. Deconvolution high resolution ESI mass spectrum of the putative VarG dimer from SEC. n proton masses have been subtracted from each (M+nH)n+ ion to yield the corresponding zero-charge mass in the deconvolved spectrum. This analysis (using 50 μg/mL of protein) revealed a major peak, with a molecular mass of 41,679 Da, and a minor peak, with a molecular mass of 83, [file pone.0184255.s005.tiff]

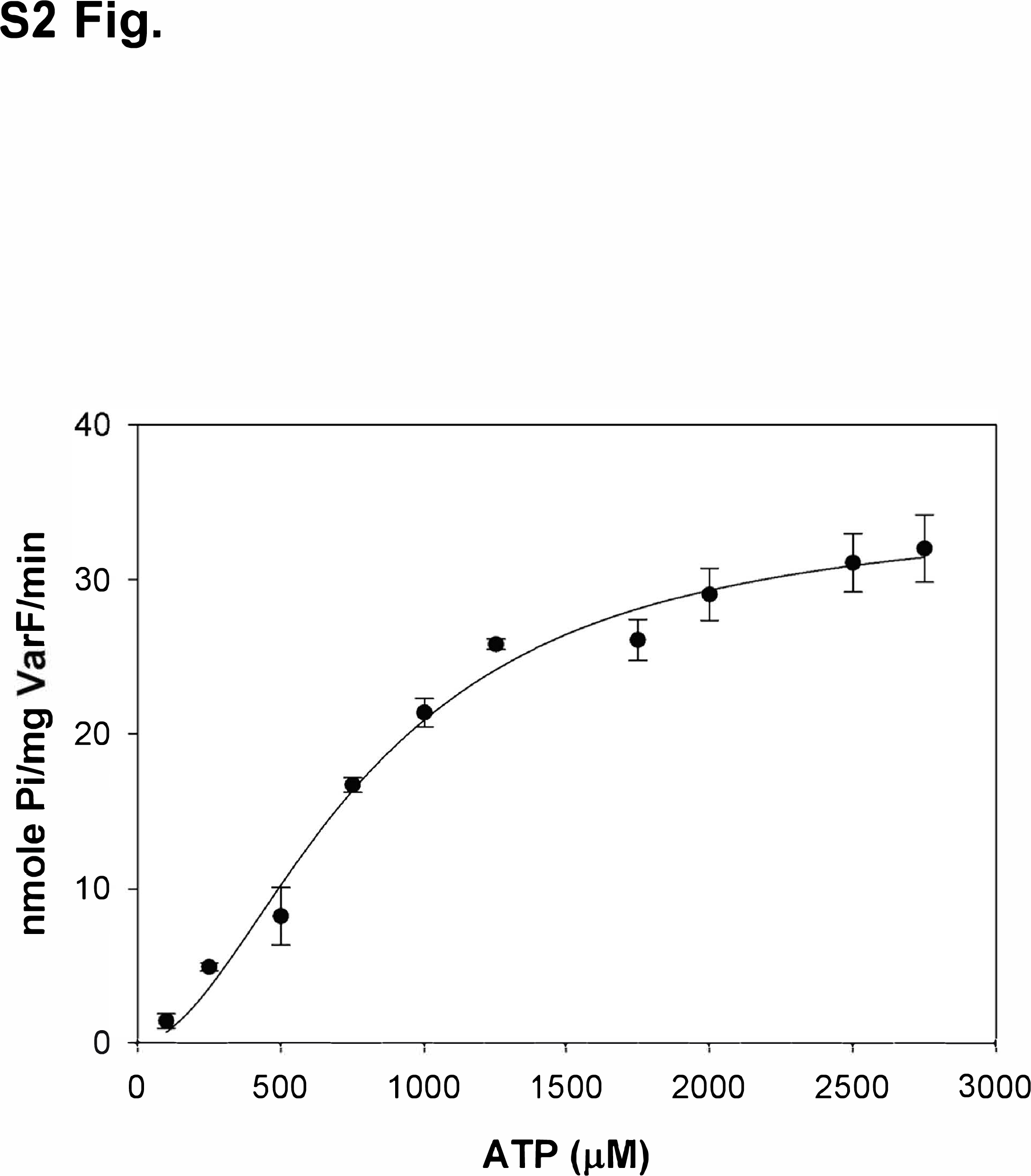

Supplement: S2 Fig — The ATPase activity of VarF was determined using a malachite green assay to monitor Pi production. The rate of ATP hydrolysis was measured as a function of the ATP concentration and the data fitted to a sigmoidal equation, indicating values for the Vmax, Km and Hill Coefficient of 34.6 (± 2.3) nmoles Pi/min/mg, 795 (± 81.2) μM and 1.87 (± 0.29), respectively. (TIFF) [file pone.0184255.s006.tiff]

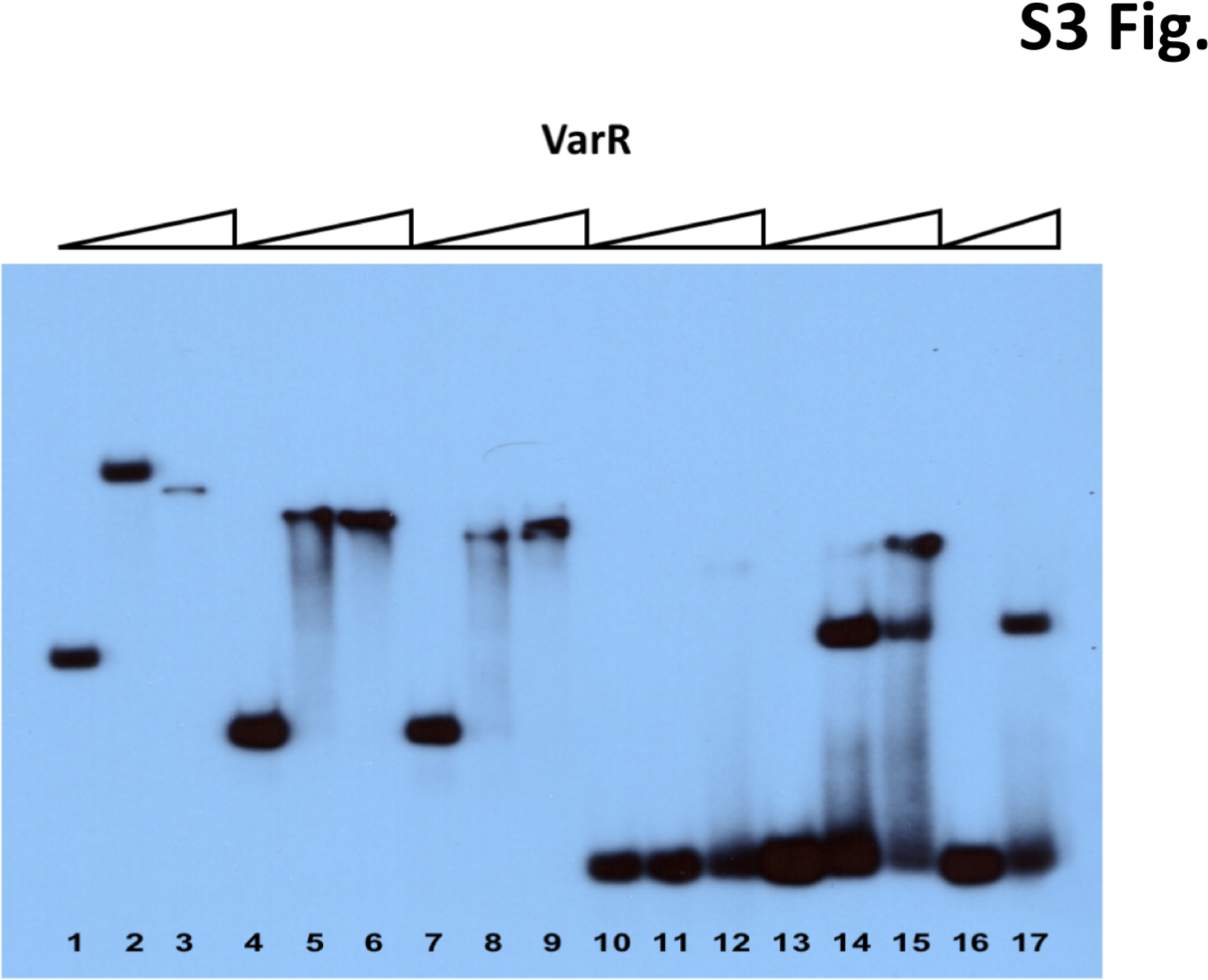

Supplement: S3 Fig — EMSA analysis of VarR binding to varR-varG IR with (0.08 ng) 302 bp, 1st and 2nd 151 bp, 1st 31 bp, 2nd 32 bp of the varR-varG IR. Titrations of VarR (0, 50, 200ng, respectively) with 0.08ng of 302 bp varR-varG IR (Lanes 1 to 3), 1st 151 bp varR-varG IR (Lanes 4 to 6), 2nd 151 bp varR-varG IR (Lanes 7 to 9), 1st 31 bp varR-varG IR (Lanes 10 to 12), and 2nd 31 bp varR-varG IR (13 to 15). Lanes 16 and 17, 0.08ng 31bp Mtr1 DNA with 0ng and 200ng MtrR, respectively (positive control). Retardation of the 302 bp, the 1st and 2nd 151 bp and the 2nd 32 bp varR-varG IR DNA fragments following incubation with 50 and 200ng VarR, respectively. However, the 1st 31bp varR-varG IR was not retarded by VarR. (TIFF) [file pone.0184255.s007.tiff]

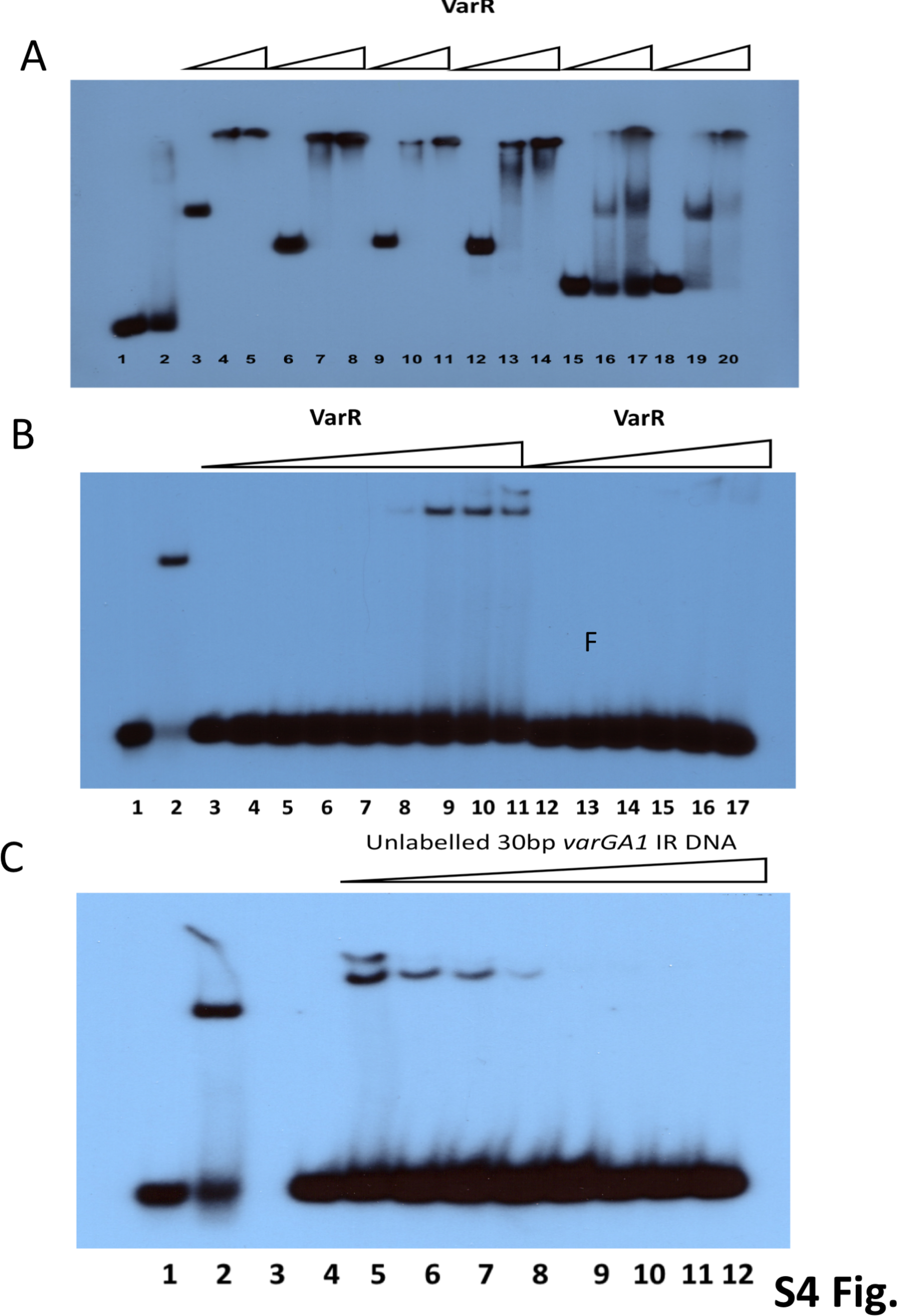

Supplement: S4 Fig — (A) EMSA analysis of VarR binding to varG-varA IR with 415 bp, 1st 207 bp, 2nd 208 bp, 176 bp, 1st and 2nd 88 bp of the varG-varA IR. Lanes 1 and 2, VarR (0 ng and 50 ng, respectively) with 0.08 ng 30 bp varR-varG IR DNA (positive control). VarR (0, 50, 100 ng, respectively) with 415 bp (Lanes 3 to 5), 1st 207 bp (Lanes 6 to 8), 2nd 208 bp (Lanes 9 to 11), 176 bp (Lanes 12 to 14), 1st 88 bp (lanes 15 to 17) and 2nd 88 bp varGA IR DNA (lanes 18 to 20). Retardations with 0.08 ng 415 bp, 1st 207 bp, 2nd 208 bp, 176 bp, 1st and 2nd 88 bp varG-varA IR DNA fragments following incubation with 0, 50, 200ng VarR, respectively, are observed. (B) EMSA using increasing titrations of VarR with 30 bp varG-varA1 IR DNA including 30 bp non-specific DNA. Lanes 1 and 2, VarR (0 and 50 ng) with 0.08 ng 30 bp varRG IR DNA (positive control). Lanes 3 to 11, titrations of VarR (0, 1.25, 2.5, 5, 10, 25, 50, 100, 200 ng, respectively) with 0.08 ng 30 bp varG-varA1 IR DNA. Lanes 12 to 17, titrations of VarR (0, 5, 10, 50, 100, 200 ng, respectively) with 0.08 ng 30 bp non-specific DNA. (C) Competitive EMSA of VarR/ 0.08 ng varG-varA1 DNA complex with unlabelled varR-varG IR DNA. Lane 1 and 2, 0 and 50 ng VarR with 0.08 ng varG-varA1 IR DNA, respectively. Lanes 3 to 12, competitive assay of 50 ng VarR/ 0.08 ng varG-varA1 IR DNA complex with titrations of unlabelled 30 bp varR-varG IR DNA (0.125, 0.25, 0.5, 1, 2, 4, 8, 16, 32, 64 ng, respectively). (TIFF) [file pone.0184255.s008.tiff]

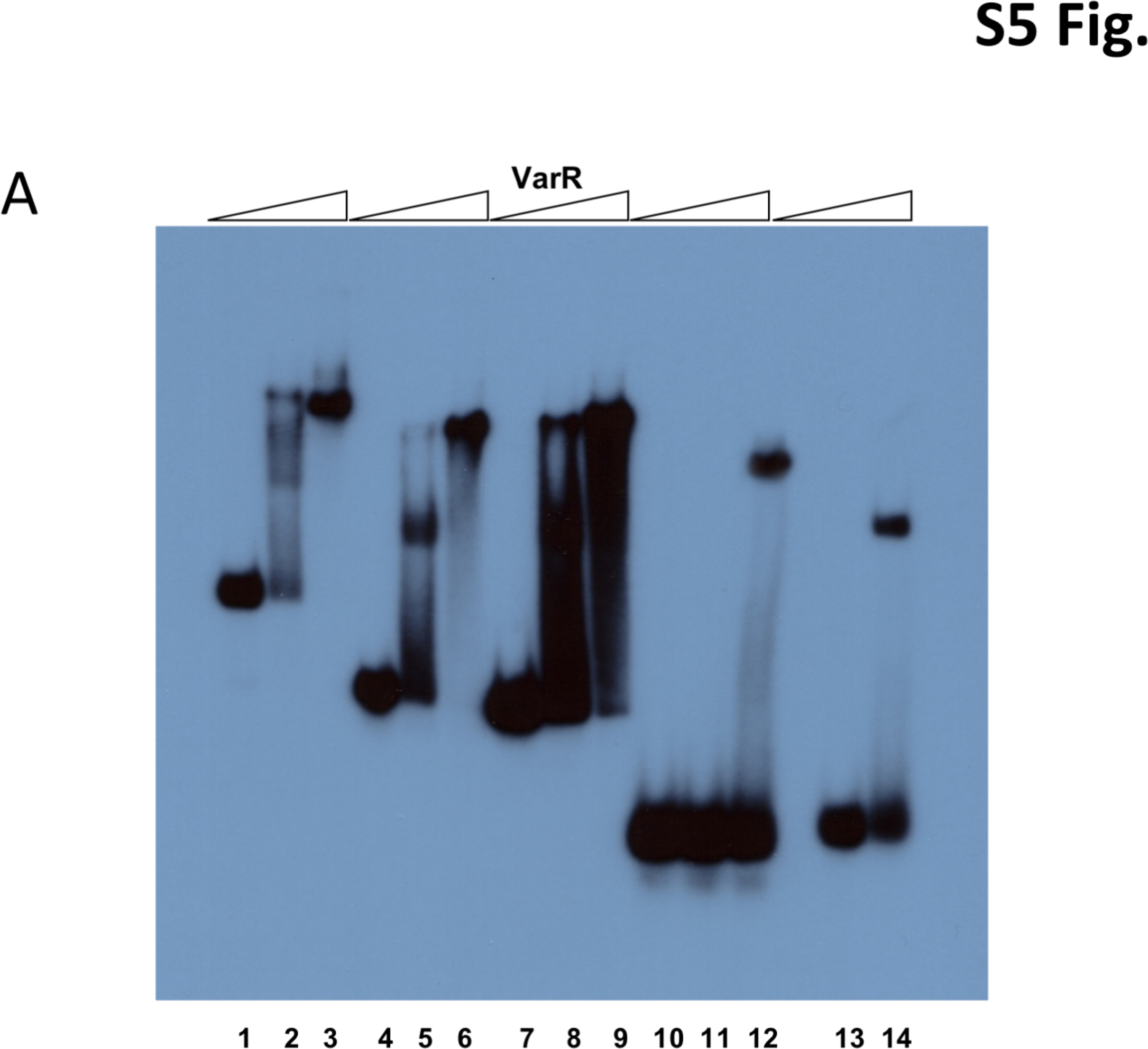

Supplement: S5 Fig — EMSA of VarR with 195 bp, 1st 97 bp, 2nd 98 bp and 25 bp of the varB-varC IR. Titrations of VarR (0, 50 and 200 ng, respectively) with 0.08 ng 195 bp varB-varC IR DNA (Lanes 1 to 3), 1st 97 bp varB-varC IR DNA (Lanes 4 to 6), 2nd 98 bp varB-varC IR DNA (Lanes 7 to 9), and 25 bp varB-varC IR DNA (Lanes 10 to 12). Lanes 13 and 14, 0 ng and 50 ng VarR with 0.08 ng 30 bp varR-varG IR DNA (positive control), respectively. Retardations with 0.08 ng 195 bp, 1st 97 bp, 2nd 98 bp and 25 bp varB-varC IR DNA fragments following incubation with 50 and 200 ng VarR are observed. (TIFF) [file pone.0184255.s009.tiff]
